# The Edinburgh Lifetime Musical Experience Questionnaire (ELMEQ): Responses and non-musical correlates in the Lothian Birth Cohort 1936

**DOI:** 10.1371/journal.pone.0254176

**Published:** 2021-07-15

**Authors:** Judith A. Okely, Ian J. Deary, Katie Overy

**Affiliations:** 1 Department of Psychology, Lothian Birth Cohort Studies, University of Edinburgh, Edinburgh, United Kingdom; 2 Reid School of Music, Edinburgh College of Art, University of Edinburgh, Edinburgh, United Kingdom; 3 Edinburgh Neuroscience, University of Edinburgh, Edinburgh, United Kingdom; Anadolu University, TURKEY

## Abstract

There is growing evidence of the potential effects of musical training on the human brain, as well as increasing interest in the potential contribution of musical experience to healthy ageing. Conducting research on these topics with older adults requires a comprehensive assessment of musical experience across the lifespan, as well as an understanding of which variables might correlate with musical training and experience (such as personality traits or years of education). The present study introduces a short questionnaire for assessing lifetime musical training and experience in older populations: the Edinburgh Lifetime Musical Experience Questionnaire (ELMEQ). 420 participants from the Lothian Birth Cohort 1936 completed the ELMEQ at a mean age of 82 years. We used their responses to the ELMEQ to address three objectives: 1) to report the prevalence of lifetime musical experience in a sample of older adults; 2) to demonstrate how certain item-level responses can be used to model latent variables quantifying experience in different musical domains (playing a musical instrument, singing, self-reported musical ability, and music listening); and 3) to examine non-musical (lifespan) correlates of these domains. In this cohort, 420 of 431 participants (97%) completed the questionnaire. 40% of participants reported some lifetime experience of playing a musical instrument, starting at a median age of 10 years and playing for a median of 5 years. 38% of participants reported some lifetime experience of singing in a group. Non-musical variables of childhood environment, years of education, childhood cognitive ability, female sex, extraversion, history of arthritis and fewer constraints on activities of daily living were found to be associated, variously, with the domains of playing a musical instrument, singing, self-reported musical ability, and music listening. The ELMEQ was found to be an effective research tool with older adults and is made freely available for future research.

## Introduction

Successful ageing is typically defined as high functioning across the domains of physical, cognitive, and mental health [1,2]. With the number of over 60-year-olds projected to reach two billion by 2050 [3], research into lifestyle factors that support healthy ageing is a priority. The potential contribution of musical experience to cognitive and mental health in later life has attracted recent scientific investigation as well as media and public interest. Learning to play a musical instrument is a cognitively stimulating activity that might increase resilience to age-related brain pathologies in later life [4]. There is evidence from reviews of mostly cross-sectional observational studies [5–7], that older people with experience playing a musical instrument (either currently or in the past) [8,9], are likely to perform better on tests of cognitive ability than their musically untrained counterparts. Others have documented a positive association between musical training and the volume of specific brain regions associated with language and memory (inferior frontal cortex and parahippocampus respectively) [10] as well as overall brain health (‘brain age’) in samples of older adults [11]. Furthermore, listening to and making music is linked to psychological and social benefits in older age [12–14].

Exploration of the relationship between musical experience and healthy ageing requires a comprehensive assessment of older adults’ lifetime musical training and experience. The nature of musical experience is highly complex and over a lifetime is perhaps even unique to each individual. Musical training and practical experience usually include training (informal or formal), practice (rehearsal or informal playing together) and performance (either playing a musical instrument or singing). Several questionnaires designed to assess an individual’s musical training and experience have been developed and are detailed in Table 1. These self-report measures quantify extent of musical training and experience, often by including questions about years of formal training, hours of practice, number of instruments played, and performance level reached. Some questionnaires define musical training as musical instrument training only [15,16], whereas others additionally include singing in this category [17–19]. In addition to quantity of musical training and experience, questionnaires can assess the characteristics of that training, including the age an individual first started learning (onset of musical training); which period(s) in their life they engaged in regular practice and performance; whether they played, rehearsed or performed regularly as part of a group, ensemble or band; and the instrument(s) they played. Reviews of the literature on musical training and cognitive ability [4,6,20], have highlighted the above listed variables as potential moderators of the association between musical training and cognitive performance. Studies that assess both the quantity and characteristics of musical training and experience will thus be well positioned to identify the conditions under which such training might be most potentially beneficial for older adults. Some studies with older adults have begun to explore some of these potential moderators [8,21].

**Table 1:** Self-report questionnaires of musical training and experience.

	Self-assessment of Musical Skills and Experience [15]	Ollen Musical Sophistication Index Questionnaire [17]	The Music USE Questionnaire [16]	Goldsmith’s Musical Sophistication Index [18]	Music Use and Background Questionnaire [19]	Edinburgh Lifetime Musical Experience Questionnaire (present study)
**Musical instrument**^1^						
Years played	✓	✓		✓		✓
Years formal training		✓		✓	✓	✓
Frequency of regular practice	✓	✓	✓	✓	✓	✓
Performance level reached		✓	✓	✓	✓	✓
Number of instruments	✓			✓		✓
Age at training onset		✓				✓
Timing of regular practice^2^						✓
Ensemble Experience^3^	✓					✓
Type of musical instrument				✓		✓
**Other domains**						
Music ability^4^	✓			✓	✓	✓
Music listening^5^	✓	✓	✓	✓	✓	✓
Musical notation^6^						✓

^1^Three questionnaires assess experience playing a musical instrument or singing [17–19].

^2^The life stage(s) when the respondent engaged in regular training, practice, and performance.

^3^Experience playing as part of a band/ensemble/orchestra.

^4^Self-reported musical ability.

^5^Music listening includes quantity, response to music, and uses of music.

^6^Three questionnaires [17–19] assess knowledge about music theory or experience composing music but do not include questions about notation specifically (e.g. ability to sight read).

Self-report questionnaires of musical training and experience can additionally assess other relevant domains of musical experience including music listening [15–19] and self-reported musical ability [15,18,19]. The latter category assesses an individual’s self-reported ability to perceive features of music such as rhythm, pitch, and melody, and/or their production abilities (e.g. their ability to sing in tune). Although musical ability is more typically assessed via behavioural tests, there is some evidence that self-reported musical ability is strongly correlated with performance on more objective behavioural tests [18]. Music listening represents a further important dimension of musical experience [16,18,19,22]. Individuals with no experience of making music can nevertheless possess a sophisticated knowledge of and receptive sensitivity to music. These attributes can depend on how an individual engages with music: the amount of time they dedicate to actively listening to music, the importance they attach to music, the extent to which they respond emotionally to music, or whether they interact with music through activities such as attending concerts or festivals, dancing or discussing music with others [18]. Questionnaires that assess music listening often measure the quantity of music listening, as well as uses of and responses to music. In addition to the self-report musical experience questionnaires outlined in Table 1, there are several others that focus specifically on various aspects of listening to music [23–30].

The musical experience questionnaires described above provide valuable methods of capturing, in detail, an individual’s musical experience. Most of these measures were designed with a specific emphasis, such as assessment of musical sophistication in the general population [18], or styles of music engagement [16]. The Edinburgh Lifetime Musical Experience Questionnaire (ELMEQ; described and used for the first time in the present study), is a musical experience questionnaire designed specifically for research on musical training and healthy ageing (with a particular focus on cognitive and brain ageing). It therefore provides a detailed, retrospective assessment of lifetime musical instrument training, including questions on the quantity and characteristics of musical training and experience (see Table 1). A further aim of the questionnaire is to assess a broader range of musical experiences than instrumental skills alone; therefore, the ELMEQ also includes questions on experience singing (assessed separately from instrumental experience), music notation reading, self-reported musical ability, and music listening (including quantity of music listening and responses to music). The ELMEQ was specifically developed for participants of the Lothian Birth Cohort 1936 (LBC1936), a multidisciplinary longitudinal cohort study that examines the nature and determinants of non-pathological cognitive ageing [31,32]. However, the questionnaire is freely available for researchers to use and offers a comprehensive tool for assessing lifetime musical experience in other samples of older adults.

The overarching aim of this paper is to provide a resource for future studies on the potential benefits of musical experience for older adults. With that intention in mind, we firstly use the ELMEQ to report on the prevalence of musical experiences (playing a musical instrument, singing, reading music notation, self-reported musical ability, and music listening) in the LBC1936 sample. Secondly, using LBC1936 participant responses, we illustrate how certain ELMEQ items that quantify experience ‘*playing a musical instrument’*, *‘experience singing’*, *‘self-reported musical ability’*, and *‘music listening’* can be used to form latent variables representing overall experience in these four domains. Such a modelling approach will be particularly useful to researchers interested in examining the potential cumulative effects of lifetime musical experience on healthy ageing outcomes and offers a means of treating musical training as a continuous rather than categorical variable. Studies on musical experience and cognitive ability in older age often compare groups of participants categorised as either “musicians” or “non-musicians” (see S1 Table for a list of these studies and further details), but such group comparisons can miss important information, since they exclude individuals with more varying levels of musical training and experience. Thirdly, little is known regarding the non-musical correlates of lifetime musical experiences among older adults. As well as being an empirical question in its own right, information regarding the characteristics of older adults with varying levels of musical experience is important for future studies in this area. This information will allow researchers to potentially control for variables that might mediate or confound the relationship between musical experience and healthy ageing outcomes.

LBC1936 participants are a deeply phenotyped cohort, with data on (but not limited to) childhood cognitive ability (assessed at age 11), childhood environment, childhood and adulthood socio-economic status (reported retrospectively in older age), as well as personality traits, disease history, and physical function, all assessed repeatedly between ages 70 and 82. This sample therefore provides a rare opportunity to examine a range of non-musical variables, from across the lifespan, that may be associated with lifetime musical experience, reported in older age. This final set of analyses tested for variables associated with the four musical experience domains *playing a musical instrument*, *singing*, *self-reported musical ability*, and *music listening*. Based on existing research with children and adults [18,33–38], we tentatively predicted that these domains would be variously positively associated with childhood environment, socio-economic resources, years of education, childhood cognitive ability, agreeableness, openness to experience, extraversion, adult neighbourhood environment, and physical health. We note that some of these non-musical variables (for instance, childhood cognitive ability) could be, at least in part, downstream consequences of musical engagement; however, it is likely that others (particularly childhood environment and socio-economic resources) may influence amounts and/or types of musical activity. The extent to which musical training and experience can influence certain life outcomes remains an active topic of research beyond the scope of the current study. To reflect this, we refer to the non-musical variables as potential “correlates” of musical experience. However, some of these variables could be treated as potential mediators of the association between musical training (or other musical experience domain) and healthy ageing in future studies.

## Materials and methods

### Participants

All participants were from the Lothian Birth Cohort 1936 (LBC1936). Most LBC1936 participants had taken part in the Scottish Mental Survey of 1947 (SMS1947) at age 11. The SMS1947 tested the cognitive ability of almost all Scottish children born in 1936 and attending school on 4 June 1947 (N = 70,805) [39]. The first Wave of the LBC1936 study was conducted between 2004 and 2007 with a sample of 1,091 participants, all born in 1936 (age mean [M] = 70) and recruited mostly from Edinburgh and the surrounding Lothians area [31,32,40]. Subsequent Waves of the LBC1936 study were conducted on a triennial basis with Waves 2, 3, and 4 taking place between 2007–2010 (N = 866; age M = 73), 2011–2013 (N = 697; age M = 76) and 2014–2017 (N = 550; age M = 79), respectively. The ELMEQ was completed by LBC1936 participants as part of Wave 5, 2017–2019 (N = 431; age M = 82).

See Taylor et al. [32] for details regarding the health and socio-economic characteristics of LBC1936 participants who left the study relative to those who returned for subsequent Waves of testing. Ethical permission was obtained from the Multi-Centre Research Ethics Committee for Scotland (Wave 1: MREC/01/0/56), the Lothian Research Ethics Committee (Wave 1: LREC/2003/2/29), and the Scotland A Research Ethics Committee (Waves 2, 3, 4 and 5: 07/MRE00/58). Written consent was obtained from participants at each Wave.

### Measures

#### Edinburgh Lifetime Musical Experience Questionnaire (ELMEQ)

The ELMEQ is a 30-item questionnaire consisting of four sections: Section 1—Musical Instruments, Section 2 –Singing, Section 3—Reading Music Notation and Section 4—Listening to Music. The aim is to capture any experience of playing a musical instrument, singing, reading notation or listening to music, regardless of genre (e.g. classical, folk, pop, rock or jazz). Most item response options consist of five or six categories representing age bracket, years of practice, hours of practice and level of expertise. Section 1 additionally includes two free-text items (list of musical instruments played and age stopped playing). Section 4 includes Likert-type scales (covering self-reported musical ability and responses to music). The ELMEQ was posted to participants as part of a larger questionnaire booklet that was completed at home prior to attending clinic visits for Wave 5 of the LBC1936 study. The first 51 participants to complete the ELMEQ were given a different version of the questionnaire that did not include the items “How important has listening to music been to you over the course of your life?” and “Would you say that you have strong emotional responses to music?”.

#### Childhood variables

Using existing LBC1936 childhood data, we included a measure of childhood cognitive ability, two measures of childhood socio-economic position (childhood environment and father’s social class) and years of education. Childhood cognitive ability was assessed using the Moray House Test No. 12 as part of the SMS1947, when participants were mostly 11 years old [39]. The Moray House Test is a test of mostly verbal reasoning although other domains of cognitive ability are represented–these are described in detail elsewhere [41]. Moray House Test scores in childhood and older age have been found to correlate significantly with scores on well-validated cognitive tests, even in very old age [42]. For the present study, Moray House Test scores were adjusted for age at time of testing and transformed to an IQ-type scale with a mean of 100 and standard deviation of 15. Childhood environment was evaluated retrospectively as part of Wave 1 of the study (at mean age 70) and included questions on the number of people living in the home, the number of rooms in the home (excluding bathroom, toilet and landings), the number of people sharing toilet facilities and whether toilet facilities were outdoors [43]. Social class was assigned based on the father’s main occupation (as reported by participants at Wave 1) using the General Register Office’s Census, 1951 Classification of Occupations [44]. Finally, years of full-time education were calculated using participants’ reported age at leaving school, any further and higher education and details of their highest qualification (all reported at Wave 1).

#### Adult and older age variables

Using existing LBC1936 data, we included measures of adult occupational social class, adult neighbourhood environmental quality, history of chronic disease and constraints on activities of daily living. At Wave 1 (mean age 70) participants reported their main occupation before retirement. Occupations were then grouped into 6 occupational social class categories ranging from professional (coded as 1) to unskilled (coded as 5) following the Classifications of Occupations system 1980 [45]. Also at Wave 1, participants were assigned a neighbourhood ‘environmental quality’ score [43], based on their home address and using the Scottish Index of Multiple Deprivation (SIMD) from 2006 [46], which ranks small geographical areas of Scotland from most deprived to least deprived based on income, employment, health, education, access to services, housing and crime. At each Wave, participants reported whether they had ever been diagnosed with diabetes, cardiovascular disease, stroke, cancer, Parkinson’s disease, dementia or arthritis. Participants also completed the Townsend scale [47], which assesses constraints on activities of daily living. To include any disease incidence or activity constraints up to age 82 (when the ELMEQ was administered) we used data reported at that age (Wave 5 of the study).

#### Personality variables

Personality was recorded at each Wave with the 50-item International Personality Item Pool (IPIP) [48]. The IPIP assesses the five personality traits described by the five-factor model: emotional stability (the opposite of neuroticism), extraversion, openness to experience (also called ‘intellect’ [49]), agreeableness, and conscientiousness. The IPIP has good internal consistency and has been validated against leading personality questionnaires, including the NEO-FFI [49]. We used personality data from Wave 5 of the study, when the ELMEQ was administered.

### Data analysis

#### Descriptive statistics

Firstly, we report responses from all participants who completed the ELMEQ. S2 Table compares participants who responded to the ELMEQ (N = 420) with participants who did not respond (N = 11) as well as those who took part in Wave 1 of the LBC1936 study at age 70 but did not take part at Wave 5 at age 82 (N = 660).

#### Measurement models

Next, using selected ELMEQ items, we modelled four latent variables quantifying the domains of: *playing a musical instrument*, *singing*, *self-reported musical ability* and *music listening*. This analysis was conducted using confirmatory factor analysis in Mplus Version 8.4 [50]. Note that some items from the ELMEQ were not included in this analysis–the domains modelled here were selected as they could be treated as latent variables, which require a minimum of three manifest indicators (the items used as indicators are highlighted in the ELMEQ questionnaire, provided in the S1 File). This analysis thus serves as an illustration of how the ELMEQ can be used, with selected responses combined to form latent variables. For consistency, we chose only ordinal items (i.e. items that had five or six response options) as indicators of each latent variable. Participants with no experience of playing a musical instrument were instructed to omit further items on that topic and proceed to the next section of the questionnaire. For the purpose of analysis, we added an additional baseline response category to each item (e.g., 0 years of formal training, 0 hours of practice, no level of music performance) for participants who reported no experience of playing a musical instrument and therefore did not respond to those items. The same approach was adopted for *singing*. The resulting distributions of responses to items related to experience playing a musical instrument and experience singing were positively skewed, because more than half of participants reported no experience. However, as these items (which consisted of five or six response categories) were treated as ordered categorical variables in the analysis, distributional assumptions about normality were not required. We examined the relationship between these four modelled forms of musical experience (*playing an instrument*, *singing*, *self-reported musical ability*, and *music listening*) by comparing nested models. The first model allowed correlations between latent factors; the second, more constrained model specified no correlations between latent factors and thus represented a simpler model in which there was no relationship between different forms of musical experience. We also tested a third, hierarchical, model in which variance shared between latent factors was modelled as a higher order latent factor representing *general musical experience*. Nested models were compared using the DIFFTEST option in Mplus. Model fit was further assessed using the comparative fit index (CFI), Tucker-Lewis index (TLI), and root-mean-square error of approximation (RMSEA) and the standardized root mean squared residual (SRMR). Following the recommendations of Schermelleh-Engel et al. [51] we consider model fit values of CFI and TLI ≥ 0.95, RMSEA ≤ 0.08, and SRMR ≤ 0.10 as indicators of acceptable fit. Areas of potential misfit were also explored by examining modification indices. Theoretically plausible parameters with modification indices ≥ 10 were considered [52].

#### Non-musical correlates of lifetime musical experience

**Fig 1:**
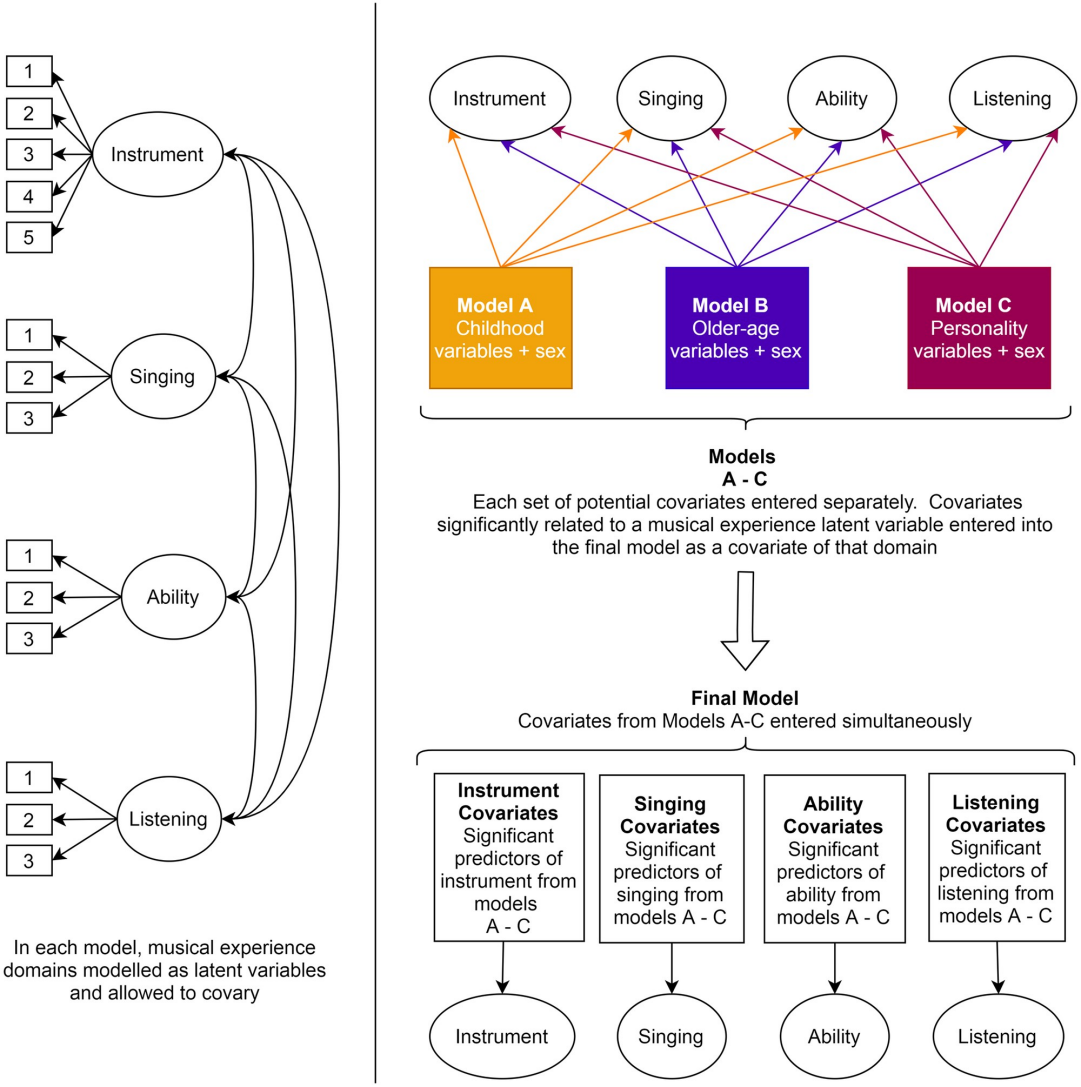
Illustration of the latent variables representing *playing a musical instrument*, *singing*, *self-reported musical ability* and *music listening* (left) and our approach to testing for associations between musical experience domains and non-musical variables (right). Ellipses represent latent variables, rectangles observed variables, single headed arrows regression paths or factor loadings and double headed arrows covariances.

Next, we examined the potential childhood, older age, and personality correlates of lifetime musical experience. We firstly ran three structural equation models (models A-C), one for each set of covariates: childhood (model A), older age (model B), and personality (model C). In each model, the four latent variables, *playing a musical instrument*, *singing*, *self-reported musical ability* and *music listening* were modelled simultaneously and regressed on the relevant set of covariates (childhood, older age, or personality covariates). Sex was additionally included as a covariate in each model. All ordinal covariate variables were treated as continuous. Residuals of the latent musical experience variables were allowed to correlate. We entered statistically significant predictors of *playing a musical instrument*, *singing*, *self-reported musical ability* and *music listening* (identified in models A-C) simultaneously into a final structural equation model. This analysis is illustrated in Fig 1.

For the confirmatory factor analysis and structural equation models (testing for non-musical correlates of musical experience), missingness of the ELMEQ items was treated using weighted least squares mean and variance adjusted (WLSMV) estimation. With WLSMV estimation, the model is conditioned on the observed exogenous covariates and cases with missing data on these variables are excluded. We therefore additionally excluded participants with missing data on childhood, older age, or personality covariate variables from the analytical sample for these analyses. S3 Table shows the number of participants with missing data on these covariate variables and S1 Fig shows a flowchart of how participants were excluded from the analytical sample. The largest proportion of missing data was observed for father’s social class (N missing = 34) followed by age 11 cognitive ability (N missing = 26). S4 Table shows the characteristics of participants included (N = 322) and excluded (N = 98) from the analytical sample. As can be seen, compared to participants included in the analytical sample, participants who were excluded were significantly less likely to report experience singing in a group and had a significantly lower SIMD score (lower neighbourhood ‘environmental quality’). Of the participants included in the analytical sample, 291 had complete data on all musical experience variables.

*Multiple comparisons*. Little has been published on the correlates of lifetime musical experience, so we treated the current analysis as exploratory and did not correct *p*-values for multiple comparisons. Our results therefore provide preliminary evidence regarding the correlates of lifetime musical experience and further confirmatory work will be needed to test whether the associations described here generalise to a wider population of older adults.

## Results

### Participant responses

Of the 431 participants who attended Wave 5 of the study, 420 (97.4%) responded to the ELMEQ (the remaining 11 did not attempt this questionnaire). There was a median of 7 missing responses per item and a range of 1–24 missing responses. Table 2 shows the number of participants with experience playing a musical instrument (N = 167, 40.3%), experience singing (N = 157, 37.8%), and experience reading music notation (N = 118, 28.8%).

**Table 2:** Participant responses to the ELMEQ (overall N = 420).

	Response	Response N	Missing/NA
Ever played a musical instrument		414	6
• No	247 (59.7%)		
• Yes	167 (40.3%)		
Currently playing		166	7/247
• No	127 (76.5%)		
• Yes	39 (23.5%)		
Ever played in a group or band		159	14/247
• No	129 (81.1%)		
• Yes	30 (18.9%)		
Ever sung in a group or choir		415	5
• No	258 (62.2%)		
• Yes	157 (37.8%)		
Any solo vocal training		156	6/258
• No	140 (89.7%)		
• Yes	16 (10.3%)		
Ever learnt to read musical notation		410	10
• No	292 (71.2%)		
• Yes	118 (28.8%)		

NA = not applicable. Percentage is based on the number of participants who responded to that question (shown in the Response N column). The last column shows the number of missing responses and the number of participants who did not respond because the question did not apply (NA).

**Fig 2:**
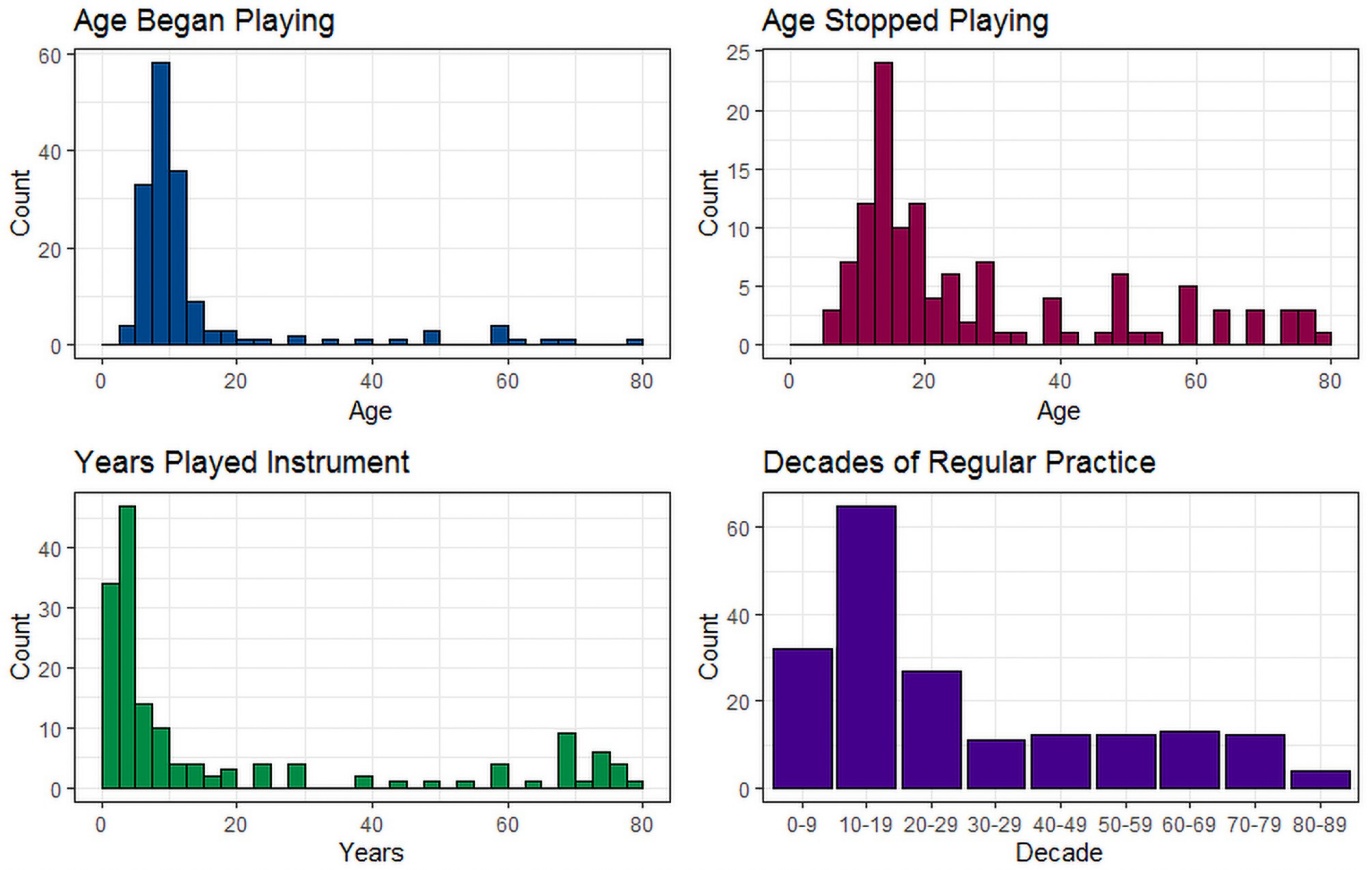
Responses to Section 1 of the ELMEQ: Musical instruments.

S5–S7 Tables show responses to items in Section 1 (Musical Instruments). Of the participants who reported learning to play a musical instrument, 115 (70.6%) learned to play only one instrument and 143 (86.1%) received formal musical training. Participants most commonly reported reaching an intermediate level of performance (N = 76, 50.0%). Fig 2 shows the age at which participants first started (Median age = 10 years, inter quartile range [IQR] = 8–12) and stopped (Median = 19, IQR = 14–40) playing a musical instrument, the number of years they played (Median = 5, IQR = 3–20), and the decades during which they practiced regularly. Some participants did report experience of playing a musical instrument in adulthood: 19 participants began playing a musical instrument at age 18 or older (range 18 to 78 years), and 46 participants reported regular practice during adulthood (between ages 20–80). Additional responses to items including ‘type(s) of musical instrument(s)’, ‘hours of practice per week’, ‘playing pieces by ear’, ‘improvising’, ‘current playing’ and ‘playing in a band or ensemble’ are detailed in S5–S7 Tables.

S8 Table shows participants’ responses to items in Section 2 (Singing). Of the participants who reported experience of singing in a group or choir, 103 (66.0%) began singing at age 12 or older; participants most commonly had 0–4 years of experience (N = 69, 44.5%) and practiced 2–3 hours per week (N = 76, 49.0%). 16 (10.3%) participants reported solo vocal training, most commonly for 2–5 years (N = 12; 80.0%).

S9 Table shows responses to Section 3 (Reading Music Notation). Of the participants who reported ever having learned to read music notation, these participants could most commonly read the treble clef (N = 97, 89.0%), 54 (45.8%) reported reaching a beginner level of sight-reading and 50 (42.4%) reported reaching an intermediate level.

S10 Table shows responses to Section 4 (Listening to Music). Participants most commonly reported listening to 2–3 hours of recorded music per week (N = 150, 36.9%), and attending 0–1 concerts or gigs per year (N = 172, 41.8%). Participants most commonly reported finding it easy (N = 172, 41.7%) or very easy (N = 173, 42.0%) to clap their hands to music, easy to dance in time to music (N = 170, 41.5%), and easy to sing a melody in tune (N = 143, 35.0%). Finally, participants most commonly reported that their parents sometimes sang songs at home (N = 125, 30.1%), that listening to music was quite important to them (N = 155, 42.5%), and that they had quite strong emotional responses to music (N = 165, 45.3%). 117 participants responded to the final free text item “Do you have any other musical experience you would like to tell us about, or any further comments?”. Qualitative analysis of the themes arising from these comments is beyond the scope of the current paper; however, commonly arising topics were musical preferences, experiences of making music at school or in church, musical family members, regrets about not having had the opportunity to learn to play a musical instrument or sing in childhood, and changes in the enjoyment of music, sometimes related to declines in hearing.

### Correlations within domains of musical experience

Correlations between indicators of *playing a musical instrument*, *singing*, *self-reported musical ability* and *music listening* are shown in S11–S13 Tables respectively. Indicators of *playing a musical instrument* were significantly positively correlated with each other (with the exception of ‘number of instruments played’ and ‘performance level reached’). Significant correlation coefficients ranged between *r* = .583 and *r* = .182. Among indicators of *singing*, ‘years of singing’ and ‘hours of practice’ were significantly positively correlated with each other (*r* = .207); ‘hours of practice’ (but not ‘years of singing’) was significantly positively correlated with ‘years of solo vocal training’ (*r* = .259). There was a significant positive correlation between all three indicators of *music listening* (ranging from *r* = .141 to *r* = .311) and between all three indicators of *self-reported musical ability* (ranging from *r* = .481 to *r* = .571).

### Measurement models

**Fig 3:**
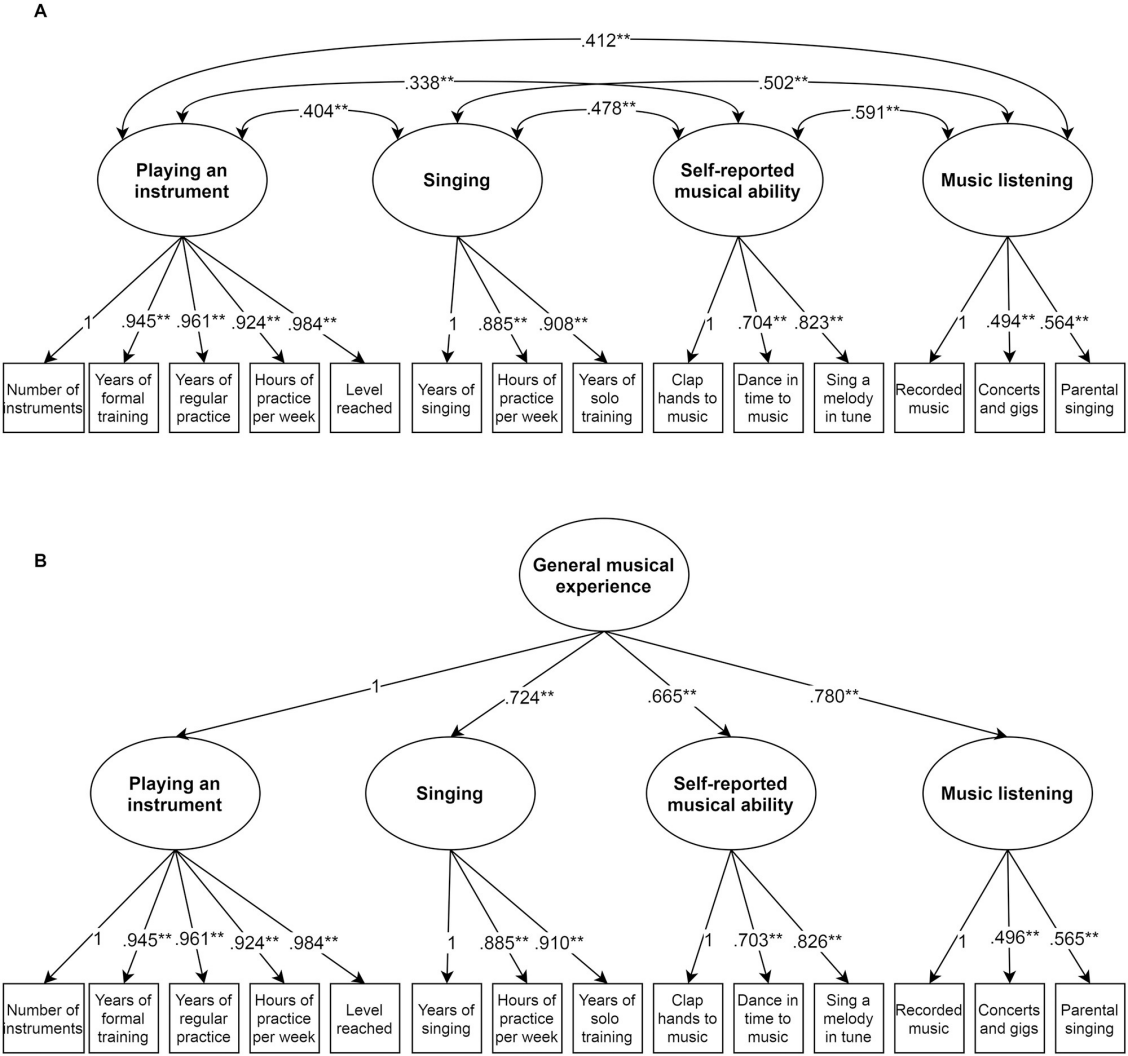
Model of musical experience latent variables and their correlations (panel A) and a higher-order model of general musical experience (panel B). Ellipses represent latent variables, rectangles observed variables, double headed arrows correlations and single headed arrows factor loadings. All path estimates are standardized. **p < .001.

This and subsequent analyses included only participants with complete data on the covariate variables (N = 322; see the Methods section for further details). Fit indices for each of the models described below are shown in Table 3. We modelled responses to the selected ELMEQ items as four latent variables representing *playing a musical instrument*, *singing*, *self-reported musical ability* and *music listening*. Initially, we compared the fit of two nested models: the first allowed correlations between the four latent variables, and the second specified no correlations. A chi-square difference test indicated that the more restricted model (no correlations between latent variables) had significantly worse fit *X*^2^(6) = 176.781, *p* < .001. The better fitting model with correlations between the latent variables is shown in panel A of Fig 3. This model provided a good fit to the data (see Table 3). Standardised factor loadings were all statistically significant and ranged between.984 for ‘performance level reached’ (indicator of *playing a musical instrument*) and.494 for ‘number of concerts/gigs per year’ (indicator of *music listening*). Correlations between the latent variables were all statistically significant (all *ps* < .001) and ranged between *r* = .338 (*playing a musical instrument* with *self-reported musical ability)* and *r* = .591 (*self-reported musical ability* with *music listening*). Inspection of modification indices for this model indicated a theoretically plausible cross-loading of the item “How easy do you find it to sing a melody in tune” on the latent variable *singing*. A modified version of the model, which specified this cross-loading, indicated a cross-loading effect of *β* = 0.384, *p* <0.001. Because the model without the cross-loading already provided a good fit to the data, we did not include this effect in the subsequent analysis.

**Table 3:** Fit indices for the three measurement models of lifetime musical experience.

Model	RMSEA (CI)	CFI	TLI	SRMR
No correlations between latent variables	0.144 (0.134, 0.155)	0.971	0.965	0.188
Correlations between latent variables	0.059 (0.046, 0.073)	0.995	0.994	0.060
Second-order *general musical experience* latent variable	0.054 (0.041, 0.068)	0.996	0.995	0.060

RMSEA = root-mean-square error of approximation, CFI = comparative fit index, TLI = Tucker-Lewis index, SRMR = standardized root mean squared residual.

The moderate to strong correlations between the latent variables suggests that their shared variance could be modelled as a higher-order factor. Including this factor representing *general musical experience* did not significantly worsen model fit *X*^2^(2) = 1.675, *p* = .433. Estimates from this model are shown in panel B of Fig 3. The loadings of the four latent variables on the higher-order *general musical experience* factor were all statistically significant and the lowest of the four loadings was.665.

### Correlates of lifetime musical experience

We tested for correlates of lifetime musical experience by adding childhood, older age, and personality variables to the model shown in panel A of Fig 3. We ran three separate models (models A-C), one for each set of variables; in each model the relevant set of potential correlates were entered simultaneously. Results from these models are shown in S14–S16 Tables. Variables significantly related to musical experience were then entered simultaneously into a final model. Estimates from this final model are shown in Table 4 and S2 Fig. A diagram of the model is also shown in S3 Fig. In this final model, *playing a musical* instrument was positively associated with a more affluent childhood environment (indicated by a lower score) (*β* = -.240, *p* = .003). *Singing* was positively associated with being female (*β* = .494, *p* = < .001), having a higher age 11 cognitive ability (*β* = .192, *p* = .019), more years of education (*β* = .195, *p* = .010) and reporting a history of arthritis (*β* = .285, *p* = .034). *Music listening* was positively associated with being female (*β* = .462, *p* = .004), having a higher age 11 cognitive ability (*β* = .182, *p* = .032) and higher extraversion (*β* = .197, *p* = .018). *Self-reported musical ability* was positively associated with being female (*β* = .325, *p* = .013), having fewer restrictions on activities of daily living (*β* = -.154, *p* = .015) and higher extraversion (*β* = .255, *p* < .001). Next, we tested whether any of the covariate variables in the final model were related to the *general musical experience* latent trait (illustrated in panel B of Fig 3). Being female (*β* = .403, *p* = .004) and higher extraversion (*β* = .190, *p* = .010) were the only variables positively related to greater *general musical experience*.

**Table 4:** Final model of lifetime musical experience and its non-musical lifespan correlates.

Experience	Covariate	*β*	95% CI	*p*
Playing an instrument	**Childhood environment**	-0.240	-0.399, -0.081	0.003
	Years of education	0.134	-0.013, 0.281	0.074
	Social class	-0.145	-0.296, 0.005	0.059
	Openness to experience^1^	0.056	-0.085, 0.197	0.439
Singing	**Sex**	0.494	0.231, 0.757	<0.001
	**Age 11 cognitive ability**	0.192	0.032, 0.353	0.019
	**Years of education**	0.195	0.046, 0.344	0.010
	Social class	-0.047	-0.213, 0.119	0.581
	**History of arthritis**	0.285	0.021,0.548	0.034
	Openness to experience^1^	0.042	-0.098, 0.182	0.555
Self-reported ability	**Sex**	0.325	0.068, 0.582	0.013
	**Activities of daily living**	-0.154	-0.279, -0.029	0.015
	**Extraversion**	0.255	0.119, 0.390	<0.001
Music listening	**Sex**	0.462	0.148, 0.777	0.004
	**Age 11 cognitive ability**	0.182	0.015, 0.348	0.032
	**Extraversion**	0.197	0.034, 0.360	0.018
	Openness to experience^1^	0.154	-0.019, 0.328	0.081

Estimates in bold are statistically significant (p < 0.05). Covariates are treated as continuous variables. Sex coded as 0 = male, 1 = female. History of disease is coded as 1 = yes, 0 = no. Lower scores on childhood environment indicate a lower level of deprivation. Lower scores on social class indicate a more professional occupation. Lower scores on the activities of daily living scale indicate fewer constraints. The table shows standardized parameter estimates. For binary covariates a different type of standardization is used which can be interpreted as a change in the dependent variable in standard deviation units when the binary covariate changes from zero to one.

^1^‘Openness to experience’ here is a personality trait, also sometimes described as ‘Intellect’ [49].

### Subsidiary analysis

In order to test for potential statistical overlap between the age 11 cognitive ability and years of education variables, we re-ran the model testing for childhood correlates of musical experience excluding the years of education variable. In this analysis, *playing a musical instrument* was additionally positively related to age 11 cognitive ability (*β* = 0.143, *p* = 0.014). In addition, we tested whether the association between *self-reported musical ability* and restrictions on activities of daily living was driven by the “how easy do you find it to dance in time to music” variable. Spearman’s rho correlations between restrictions on activities of daily living score and the three indicators of *self-reported musical ability* (‘singing in tune’, ‘clap to music’ and ‘dance in time to music’) revealed that only the ‘dance in time to music’ item was significantly negatively correlated with restrictions on activities of daily living (*r*_*s*_ = -0.125, *p* = 0.027).

## Discussion

The ELMEQ was found to be an effective tool for assessing the quantity and characteristics of an older adult’s lifetime experience playing a musical instrument, as well as musical experiences including singing, self-reported musical ability, listening to music and reading music notation. The ELMEQ was completed by participants at home, had an excellent response rate (97%) and little missing data (see S5–S10 Tables for details), suggesting that it was acceptable to participants. Our results indicate that only a small proportion of participants (9%) currently played a musical instrument; however, 40% had some lifetime experience of playing and a similar proportion (38%) reported experience of singing in a group. Selected items from the ELMEQ were used to model four domains of musical experience quantifying *playing a musical instrument*, *singing*, *self-reported musical ability* and *music listening*. There was a significant positive relationship between each of these domains of musical experience. Significant non-musical correlates of greater experience across the four musical experience domains included a more affluent childhood environment, more years of education, higher childhood cognitive ability, female sex, a positive history of arthritis, higher extraversion and fewer constraints on activities of daily living; these results are discussed below.

Some other cohort studies of older adults have reported the percentage of participants who currently play an instrument and/or sing, with estimates ranging from 4% in the Bronx Aging Study (mean age = 79) [53] to 25% in the Longitudinal Aging Study Amsterdam (mean age = 74) [54]. Results from the Scottish Household Survey 2018 [55] indicate that around 4% of people living in Scotland aged 75 and over currently play a musical instrument. The present study provides a broader picture of older adults’ music making experiences including their lifetime history of playing a musical instrument. Our findings support previous work suggesting that only a small proportion of older adults continue to play a musical instrument in older age, but also show that substantially more have some lifetime experience of playing (9% and 40% in this LBC1936 cohort, respectively). LBC1936 participants mostly learned to play a musical instrument in childhood and practiced for a median of 5 years in total. These estimates are largely in line with those reported by other studies conducted with samples of adults and older people with a history of musical training [8,19,56]. Thus, with a view to studying the potential impact of musical training and experience on outcomes in older age, it is important to note that, in the general population, exposure to musical instrumental training typically occurs only for a short period of time early in life. Nevertheless, a small number of participants in the LBC1936 cohort reported regularly training and practicing in adulthood (N = 46), and some participants only began musical training in adulthood or older age (N = 18). Future studies could use the ELMEQ to investigate whether the timing of regular practice or age at training onset moderate the relationship between musical training and healthy cognitive ageing, or other outcomes.

We found that selected items from the ELMEQ could be used as indicators of four latent variables representing the domains of *playing a musical instrument*, *singing*, *self-reported musical ability* and *music listening*. Model comparisons revealed that all four domains of musical experience were moderately to strongly correlated, and that these correlations could be represented by a hierarchical model in which a *general musical experience* latent variable accounted for shared variance between each musical experience domain. These findings are in line with those of two previous studies [18,19] that report strong correlations between various domains of musical experience, some of which overlap with the domains used here (including *playing a musical instrument* and *self-reported musical ability*). Overall, these findings provide evidence in favour of a “general musical experience” or “musicality” trait that describes a person’s level of musical engagement across a range of domains. Nevertheless, it is possible that different forms of musical experience are differentially related to healthy ageing outcomes. For instance, in a cross-sectional study of older adults, Mansens et al. [54] found that a group of participants who only played a musical instrument performed better on a test of processing speed than a group of participants who only sang. Furthermore, different elements of musical experience (such as rhythm and melody processing) involve distinct neural components [57,58]. Therefore, researchers may choose to examine the relationship between musical experience and healthy ageing from a more fine-grained perspective. The ELMEQ questionnaire allows for such a fine-grained approach while also gathering information on related musical experiences that are likely to correlate.

Greater experience *playing a musical instrument* was significantly related to a more affluent childhood environment. This finding corroborates previous reports of a positive association between socio-economic circumstances and instrumental training reported in childhood [33,34], and indicates that this association extends to lifetime experience of musical training, reported in older age. It is likely that this relationship in part reflects the financial barriers to music participation in childhood including less access to musical training in less affluent areas and schools [59]. We did not observe a significant association between *playing a musical instrument* and childhood cognitive ability (in multivariate analysis additionally controlling for childhood socio-economic resources, years of education, and sex). However, including years of education and cognitive ability in the same model may have resulted in statistical over-adjustment, as these variables are strongly positively correlated [60]. In subsidiary analysis, we found that childhood cognitive ability was positively related to experience playing a musical instrument when years of education was excluded from the model. This result may support previous observational and experimental studies documenting a positive association between musical training and cognitive ability [20,36,61–64] however, it is also plausible that education mediates the relationship between cognitive ability and playing a musical instrument. That is, individuals with a higher cognitive ability at age 11 might spend more years in education, which in turn might increase opportunities for musical training.

Greater experience *singing* was positively associated with more years of education and a higher childhood cognitive ability, in agreement with previous reports regarding the correlates of musical training [33,35,36,38]. The positive relationship between *singing* and female sex observed in the present study, has also been documented elsewhere [55,65]. It is unclear why history of arthritis was positively related to *singing*, this may be a chance finding; alternatively, it could indicate that singing is more accessible to people with arthritis than other forms of musical engagement that rely on fine motor skills such as playing an instrument.

In further agreement with previous research [35,36] we found that *playing a musical instrument* and *singing* were positively related to the personality trait openness to experience (in models additionally controlling for the other four personality traits and sex). However, these associations did not survive adjustment for childhood and older age variables, in the final model. We did not replicate the association between agreeableness or extraversion and experience playing a musical instrument reported by others [18,37]. However, these prior studies did not mutually adjust for all five personality traits (which are substantially intercorrelated) in the same model, as we did here.

*Self-reported musical ability* (which included an item on singing in tune) was positively related to female sex; this relationship potentially relates to the higher percentage of women reporting experience singing (61%). Additionally, lower *self-reported musical ability* was related to poorer physical function (a higher ‘activities of daily living’ score), although subsidiary analysis indicated that this finding was driven by the ‘dance in time to music’ item. Researchers interested in musical abilities that are independent of physical function may wish to omit this item, or at least take this finding into consideration. Finally, the positive relationship between extraversion and *self-reported musical ability* in our study corroborates another report of a similar correlation between extraversion and self-reported music perception and singing abilities in young adults [18]. It should however be acknowledged that self-assessed measures can be sensitive to factors other than actual ability level, such as the respondent’s confidence or the effect of social desirability.

*Music listening* was positively associated with being female. This effect was unexpected as several recent reports on current trends in music consumption (among teenage or adult participants from the US, Spain and the UK) indicate that men typically spend more time listening to music than women [66–68]. It is possible that the opposite effect observed in the LBC1936 is specific to this older cohort, although another study with a sample of 99 older adults (aged between 65–90) found no difference in time spent listening to music between men and women [69]. We also observed a positive relationship between *music listening* and childhood cognitive ability. This result fits with some previous research with the LBC1936 sample which documented a positive association between childhood cognitive ability and participation in sociocultural activities (including going to concerts) at age 70. The authors of that study suggest that individuals with a higher cognitive ability might seek out cognitively stimulating pastimes. Finally, in further agreement with previous work [18], we observed a positive association between the personality trait extraversion and *music listening*.

The non-musical variables that we found to be associated with lifetime musical experience (childhood cognitive ability, childhood environment, years of education, personality traits, and physical health status) are themselves related to various aspects of healthy ageing including physical, psychological, and cognitive health in later life [43,70–75]. These covariates should therefore be included as potential confounding or mediating variables (where possible) in future studies testing for associations between musical experience and healthy aging. A further issue that could be considered in future studies is whether associations might be driven by shared genetic factors. Research with pairs of twins indicates that frequency of music practice and music accomplishment might be partly genetically influenced [76], and there is some evidence to suggest that genetic factors may account for some of the shared variance between hours of music practice and general cognitive ability [77].

Strengths of the present study include the relatively large sample size, narrow age range of participants and the detailed data available regarding participants’ childhood circumstances and cognitive ability as well as demographic, health and personality characteristics in older age. The study’s limitations should also be considered. Firstly, owing to the novelty of the research topic, we treated this as an exploratory study and did not correct *p*-values for multiple comparisons. The associations described in this study should therefore be replicated in further confirmatory analysis. Furthermore, LBC1936 participants are from a limited geographical area, and are all white British (mostly Scottish) in background. The LBC1936 sample is characterised by higher levels of healthiness, socio-economic recourses, and cognitive ability compared with the general population. Due to the over-representation of individuals from higher socio-economic groups in this sample, the proportion of older adults with experience of playing a musical instrument in the general Scottish population may be lower than the 40% reported here. On the other hand, studies have illustrated that correlates of musical experience such as socio-economic factors may be less strongly related to music making in particular regions where there is a strong community tradition of musical engagement [18]. Therefore, the correlates of musical experience identified in the LBC1936 sample may not be generalizable to other regions of the UK, or indeed to populations in other countries [78]. The ELMEQ does not cover areas such as music technology, writing/composing music or dancing. We also recognise that strong, positive musical identities can be formed through musical preferences and social relationships around music, rather than directly through skills and experiences [79] something that we did not examine in this questionnaire. In addition, some potential non-musical correlates were not recorded as part of the LBC1936 study and were therefore not included in the analysis. These include parents’ characteristics such as personality, cognitive ability, and musical background [37]. Finally, it should also be noted that the ELMEQ relies on participants having a reasonably accurate memory of their lifetime musical experiences. However, retrospective measures of lifetime activity are commonly used in observational studies of ageing and have good validity in the case of lifetime history of smoking [80] and physical activity [81].

### Future directions

Numerous longitudinal cohort studies of older adults have been established internationally. Many of these observational studies include questions about past and current leisure activities, repeated assessments of cognitive ability and psychological wellbeing. However, only a few of these studies currently collect information about musical training; furthermore, studies that do include such assessments (see the Mayo Clinic Study of Aging [82], the Longitudinal Aging Study Amsterdam [83], and the Swedish National Study on Ageing and Care [84]) tend to focus on current musical activities rather than past musical training and experience. The addition of a broader musical experience assessment to large, established cohort studies (as we did here with the LBC1936) could provide a cost-effective and powerful means of progressing research on musical experience and healthy ageing. This approach would allow researchers to test for associations between musical experience and a variety of outcomes including cognitive or brain ageing as well as psychological and social wellbeing. Such research might be particularly effective if assessment of musical experience and training is harmonised across multiple cohort studies (thus allowing direct comparisons or replications across multiple studies). The ELMEQ, which was specifically designed for a longitudinal cohort study of older adults, could be a particularly useful tool in this context.

Findings from the present study and the ELMEQ itself could be valuable in other contexts too. As the body of evidence documenting the potential benefits of musical training and experience grows, from improved psychological wellbeing to more positive cognitive development and cognitive ageing [4–7,13,14,64] so does the argument for widening access to participation in musical activities. Of course, there is also a longstanding and perhaps even stronger argument for the intrinsic value of musical training and experience, which can offer the development of musical skills, self-expression, creativity, social and cultural engagement and indeed musical careers [85]. Regardless of motivations, and in addition to the need for appropriate funding, achieving the goal of widening opportunities will be supported by an understanding of the determinants of and barriers to musical engagement in the general population. In the present study, we found that individuals who were male, had fewer socio-economic resources, fewer years of education or had a lower childhood cognitive ability, were less likely to report certain musical experiences, such as playing a musical instrument or singing. These results in combination with earlier findings [18,33–38] could help to identify groups who are less likely to participate in musical activities and who may benefit from additional support to do so. This information is complemented by findings from studies using qualitative methods, which have explored the perspectives of less musically experienced individuals and identified some of the barriers to beginning or continued musical participation [86–88]. A further finding from the qualitative research literature is that, in order to be successful, music making opportunities should be matched to the individual’s ambitions and prior musical experiences [86]. A more practical application of the ELMEQ could involve administering the questionnaire to individuals who might be interested in musical participation (particularly later in life) and using their responses to match them to an appropriate musical opportunity.

On a final note, we have included an updated version of the ELMEQ in the S1 File. The ELMEQ may be freely used by researchers; no permission is needed from the present authors to use it, though we are happy to be contacted about it, and we welcome translations (with appropriate checks, including back-translation). This version includes some minor changes to the original questionnaire (used in the present study) that were made following our analysis of participants’ responses. These updates are also detailed in the S1 File.

### Conclusions

The ELMEQ provides a new tool for assessing lifetime musical experience which could facilitate future studies involving older adults. The questionnaire was designed to be completed by a cohort of adults in their 80s and provides a comprehensive assessment of musical experience that moves beyond categorising individuals as musicians and non-musicians. The current study documents the prevalence of lifetime musical experiences in a Scottish sample of older adults. Our analysis illustrates how selected ELMEQ items can be used to create composite scores quantifying lifetime experience playing a musical instrument, singing, self-reported musical ability and music listening. Other items provide detailed information regarding the characteristics of musical experiences that allow for future testing of refined and specific predictions (for instance, whether early onset of instrumental training, or learning an instrument in later life are associated with cognitive or other outcomes in older age). Furthermore, our results identify some of the non-musical lifespan correlates of musical experience. We hope that this work will inform future assessment of lifetime musical experience and research into its potential relationship with healthy ageing.

## Supporting information

S1 Fig Flowchart showing the number of participants excluded from the analytical sample for the factor analysis and structural equation models. (DOCX)

S2 Fig Non-musical variables associated with the musical experience domains. (DOCX)

S3 Fig Diagram of the final model of lifetime musical experience and its lifespan correlates. (DOCX)

S1 Table Categories of musical experience used in observational studies of cognitive ageing or dementia risk. (DOCX)

S2 Table Characteristics of participants who responded to the ELMEQ and of those who did not. (DOCX)

S3 Table Characteristics of participants who responded to the ELMEQ and number of participants with missing data on the non-musical covariate variables. (DOCX)

S4 Table Characteristics of participants included and excluded from the analytical sample due to missing data points. (DOCX)

S5 Table Responses to Section 1: Experience playing a music instrument. (DOCX)

S6 Table Responses to Section 1: Experience playing multiple instruments. (DOCX)

S7 Table Responses to Section 1: Experience playing in a band or ensemble. (DOCX)

S8 Table Responses to Section 2: Experience singing. (DOCX)

S9 Table Responses to section 3: Reading musical notation. (DOCX)

S10 Table Responses to Section 4: Listening to music. (DOCX)

S11 Table Correlations between indicators of *playing a musical instrument*. (DOCX)

S12 Table Correlations between indicators of *singing*. (DOCX)

S13 Table Correlations between responses to Section 4: *Listening to music*. (DOCX)

S14 Table Childhood correlates of musical experience. (DOCX)

S15 Table Older age correlates of musical experience. (DOCX)

S16 Table Personality correlates of musical experience. (DOCX)

S1 File Updated version of the Edinburgh Lifetime Musical Experience Questionnaire (ELMEQ). (DOCX)

## References

[1] Depp CA, Jeste DV. Definitions and predictors of successful aging: a comprehensive review of larger quantitative studies. Am J Geriatr Psychiatry. 2006;14: 6–20. doi: 10.1097/01.JGP.0000192501.03069.bc 16407577

[2] Rowe JW, Kahn RL. Successful Aging. The Gerontologist. 1997;37: 433–440. doi: 10.1093/geront/37.4.433 9279031

[3] Vojak F. Ageing, Longevity and Demographic Change: A Factpack of Statistics from the International Longevity Centre-UK. ILC: London; 2013. https://ilcuk.org.uk/wp-content/uploads/2018/10/ILC-UK_Factpack.pdf.

[4] Sutcliffe R, Du K, Ruffman T. Music Making and Neuropsychological Aging: A Review. Neurosci Biobehav Rev. 2020;113: 479–491. doi: 10.1016/j.neubiorev.2020.03.026 32302600

[5] Roman-Caballero R, Arnedo M, Trivino M, Lupianez J. Musical practice as an enhancer of cognitive function in healthy aging-A systematic review and meta-analysis. PloS One. 2018;13. doi: 10.1371/journal.pone.0207957 30481227 PMC6258526

[6] Schneider CE, Hunter EG, Bardach SH. Potential Cognitive Benefits From Playing Music Among Cognitively Intact Older Adults: A Scoping Review. J Appl Gerontol. 2018;38: 1763–1783. doi: 10.1177/0733464817751198 29361873

[7] Walsh S, Causer R, Brayne. Does playing a musical instrument reduce the incidence of cognitive impairment and dementia? A systematic review and meta-analysis. Aging Ment Health. 2019. doi: 10.1080/13607863.2019.1699019 31814445

[8] Hanna-Pladdy B, Gajewski B. Recent and past musical activity predicts cognitive aging variability: direct comparison with general lifestyle activities. Front Hum Neurosci. 2012;6: 198. doi: 10.3389/fnhum.2012.00198 22833722 PMC3400047

[9] Strong JV, Midden A. Cognitive differences between older adult instrumental musicians: Benefits of continuing to play. Psychol Music. 2020;48: 67–83. doi: 10.1177/0305735618785020

[10] Chaddock-Heyman L, Loui P, Weng TB, Weisshappel R, McAuley E, Kramer AF. Musical training and brain volume in older adults. Brain Sci. 2021;11: 50. doi: 10.3390/brainsci11010050 33466337 PMC7824792

[11] Rogenmoser L, Kernbach J, Schlaug G, Gaser C. Keeping brains young with making music. Brain Struct Funct. 2018;223: 297–305. doi: 10.1007/s00429-017-1491-2 28815301

[12] Park A-L. Can musical activities promote healthy ageing? Int J Emerg Ment Health Hum Resil. 2015;17: 258–261.

[13] Perkins R, Williamon A. Learning to make music in older adulthood: A mixed-methods exploration of impacts on wellbeing. Psychol Music. 2014;42: 550–567. doi: 10.1177/0305735613483668

[14] Tymoszuk U, Perkins R, Spiro N, Williamon A, Fancourt D. Longitudinal associations between short-term, repeated, and sustained arts engagement and well-being outcomes in older adults. J Gerontol Ser B. 2020;75: 1609–1619. doi: 10.1093/geronb/gbz085 31287550 PMC7424284

[15] Cuddy LL, Balkwill L-L, Peretz I, Holden RR. Musical difficulties are rare: A study of “tone deafness” among university students. Ann N Y Acad Sci. 2005;1060: 311–324. doi: 10.1196/annals.1360.026 16597781

[16] Chin T-C, Rickard NS. The music USE (MUSE) questionnaire: An instrument to measure engagement in music. Music Percept Interdiscip J. 2012;29: 429–446. doi: 10.1525/mp.2012.29.4.429

[17] Ollen JE. A criterion-related validity test of selected indicators of musical sophistication using expert ratings. PhD Thesis, The Ohio State University. 2006. http://rave.ohiolink.edu/etdc/view?acc_num=osu1161705351.

[18] Müllensiefen D, Gingras B, Musil J, Stewart L. The musicality of non-musicians: an index for assessing musical sophistication in the general population. PloS One. 2014;9: e89642. 2016-17512-001. doi: 10.1371/journal.pone.0089642 24586929 PMC3935919

[19] Chin T-C, Coutinho E, Scherer KR, Rickard NS. MUSEBAQ: A modular tool for music research to assess musicianship, musical capacity, music preferences, and motivations for music use. Music Percept Interdiscip J. 2018;35: 376–399. doi: 10.1525/mp.2018.35.3.376

[20] Swaminathan S, Schellenberg EG. Music training. Cognitive training. Cham, Switzerland: Springer; 2021. pp. 307–318.

[21] Hanna-Pladdy B, MacKay A. The relation between instrumental musical activity and cognitive aging. Neuropsychology. 2011;25: 378. doi: 10.1037/a0021895 21463047 PMC4354683

[22] Hallam S, Prince V. Conceptions of musical ability. Res Stud Music Educ. 2003;20: 2–22. doi: 10.1177/1321103X030200010101

[23] Rentfrow PJ, Gosling SD. The do re mi’s of everyday life: the structure and personality correlates of music preferences. J Pers Soc Psychol. 2003;84: 1236. doi: 10.1037/0022-3514.84.6.1236 12793587

[24] Werner PD, Swope AJ, Heide FJ. The music experience questionnaire: Development and correlates. J Psychol. 2006;140: 329–345. doi: 10.3200/JRLP.140.4.329-345 16967740

[25] Chamorro-Premuzic T, Furnham A. Personality and music: Can traits explain how people use music in everyday life? Br J Psychol. 2007;98: 175–185. doi: 10.1348/000712606X111177 17456267

[26] Kreutz G, Schubert E, Mitchell LA. Cognitive styles of music listening. Music Percept Interdiscip J. 2008;26: 57–73. doi: 10.1525/mp.2008.26.1.57

[27] McDonald C, Stewart L. Uses and functions of music in congenital amusia. Music Percept Interdiscip J. 2008;25: 345–355. doi: 10.1525/mp.2008.25.4.345

[28] Saarikallio S, Gold C, McFerran K. Development and validation of the H ealthy-U nhealthy M usic S cale. Child Adolesc Ment Health. 2015;20: 210–217. doi: 10.1111/camh.12109 26726295 PMC4690158

[29] Mas-Herrero E, Marco-Pallares J, Lorenzo-Seva U, Zatorre RJ, Rodriguez-Fornells A. Individual differences in music reward experiences. Music Percept Interdiscip J. 2013;31: 118–138. doi: 10.1525/mp.2013.31.2.118

[30] Saarikallio S. Music in mood regulation: Initial scale development. Music Sci. 2008;12: 291–309. doi: 10.1177/102986490801200206

[31] Deary IJ, Gow AJ, Pattie A, Starr JM. Cohort Profile: The Lothian Birth Cohorts of 1921 and 1936. Int J Epidemiol. 2012;41: 1576–1584. doi: 10.1093/ije/dyr197 22253310

[32] Taylor AM, Pattie A, Deary IJ. Cohort Profile Update: The Lothian Birth Cohorts of 1921 and 1936. Int J Epidemiol. 2018. doi: 10.1093/ije/dyy022 29546429 PMC6124629

[33] Elpus K, Abril CR. Who enrolls in high school music? A national profile of US students, 2009–2013. J Res Music Educ. 2019;67: 323–338. doi: 10.1177/0022429419862837

[34] Kinney DW. Selected nonmusic predictors of urban students’ decisions to enroll and persist in middle school band programs. J Res Music Educ. 2010;57: 334–350. doi: 10.1177/0022429409350086

[35] Swaminathan S, Schellenberg EG. Musical competence is predicted by music training, cognitive abilities, and personality. Sci Rep. 2018;8: 1–7. 29907812 10.1038/s41598-018-27571-2 PMC6003980

[36] Corrigall KA, Schellenberg EG, Misura NM. Music training, cognition, and personality. Front Psychol. 2013;4: 222. doi: 10.3389/fpsyg.2013.00222 23641225 PMC3639373

[37] Corrigall KA, Schellenberg EG. Predicting who takes music lessons: Parent and child characteristics. Front Psychol. 2015;6: 282. doi: 10.3389/fpsyg.2015.00282 25852601 PMC4371583

[38] Alegrado A, Winsler A. Predictors of taking elective music courses in middle school among low-SES, ethnically diverse students in Miami. J Res Music Educ. 2020;68: 5–30. doi: 10.1177/0022429420908282

[39] Scottish Council for Research in Education. The Trend of Scottish Intelligence. London: University of London Press; 1949.

[40] Deary IJ, Gow AJ, Taylor MD, Corley J, Brett C, Wilson V, et al. The Lothian Birth Cohort 1936: a study to examine influences on cognitive ageing from age 11 to age 70 and beyond. BMC Geriatr. 2007;7: 28. doi: 10.1186/1471-2318-7-28 18053258 PMC2222601

[41] Deary IJ, Whiteman MC, Starr JM, Whalley LJ, Fox HC. The impact of childhood intelligence on later life: following up the Scottish mental surveys of 1932 and 1947. J Pers Soc Psychol. 2004;86: 130. doi: 10.1037/0022-3514.86.1.130 14717632

[42] Deary IJ, Pattie A, Starr JM. The stability of intelligence from age 11 to age 90 years: the Lothian birth cohort of 1921. Psychol Sci. 2013;24: 2361–2368. doi: 10.1177/0956797613486487 24084038

[43] Johnson W, Corley J, Starr JM, Deary IJ. Psychological and physical health at age 70 in the Lothian Birth Cohort 1936: Links with early life IQ, SES, and current cognitive function and neighborhood environment. Health Psychol. 2011;30: 1. doi: 10.1037/a0021834 21299289

[44] Office, General. Register. Census 1951: Classification of occupations. London: Her Majesty’s Stationary Office; 1956.

[45] Office of Population Censuses and Surveys. Classification of occupations 1980. London: Her Majesty’s Stationary Office; 1980.

[46] Scottish Executive. Scottish Index of Multiple Deprivation 2006 Technical Report. Edinburgh: Office of the Chief Statistician, Scottish Executive; 2006.

[47] Townsend P. Poverty in the United Kingdom: a survey of household resources and standards of living. Univ of California Press; 1979.

[48] Goldberg LR. A broad-bandwidth, public domain, personality inventory measuring the lower-level facets of several five-factor models. Personal Psychol Eur. 1999;7: 7–28.

[49] Gow AJ, Whiteman MC, Pattie A, Deary IJ. Goldberg’s ‘IPIP’Big-Five factor markers: Internal consistency and concurrent validation in Scotland. Personal Individ Differ. 2005;39: 317–329. doi: 10.1016/j.paid.2005.01.011

[50] Muthén LK, Muthén B. Mplus User’s Guide. Eighth Edition. Los Angeles: Muthén & Muthén; 1998. https://www.statmodel.com/html_ug.shtml.

[51] Schermelleh-Engel K, Moosbrugger H, Müller H. Evaluating the fit of structural equation models: Tests of significance and descriptive goodness-of-fit measures. Methods Psychol Res Online. 2003;8: 23–74. 2003-08119-003.

[52] Brown TA. Confirmatory factor analysis for applied research. New York: Guilford publications; 2015.

[53] Verghese J, Lipton RB, Katz MJ, Hall CB, Derby CA, Kuslansky G, et al. Leisure activities and the risk of dementia in the elderly. N Engl J Med. 2003;348: 2508–2516. doi: 10.1056/NEJMoa022252 12815136

[54] Mansens D, Deeg DJH, Comijs HC. The association between singing and/or playing a musical instrument and cognitive functions in older adults. Aging Ment Health. 2017; 1–8. doi: 10.1080/13607863.2017.1328481 28521542

[55] SHS Project Team. Scotland’s People Annual Report 2018. Edinburgh: Scottish Government; 2019.

[56] Gooding LF, Abner EL, Jicha GA, Kryscio RJ, Schmitt FA. Musical training and late-life cognition. Am J Alzheimers Dis Other Demen. 2014;29: 333–343. doi: 10.1177/1533317513517048 24375575 PMC4074275

[57] Koelsch S, Siebel WA. Towards a neural basis of music perception. Trends Cogn Sci. 2005;9: 578–584. doi: 10.1016/j.tics.2005.10.001 16271503

[58] Limb CJ. Structural and functional neural correlates of music perception. Anat Rec Part Discov Mol Cell Evol Biol Off Publ Am Assoc Anat. 2006;288: 435–446. doi: 10.1002/ar.a.20316 16550543

[59] Albert DJ. Socioeconomic status and instrumental music: What does the research say about the relationship and its implications? Update Appl Res Music Educ. 2006;25: 39–45. doi: 10.1177/87551233060250010105

[60] Batty GD, Deary IJ, Macintyre S. Childhood IQ in relation to risk factors for premature mortality in middle-aged persons: the Aberdeen Children of the 1950s study. J Epidemiol Community Health. 2007;61: 241–247. doi: 10.1136/jech.2006.048215 17325403 PMC2652919

[61] Degé F, Kubicek C, Schwarzer G. Music lessons and intelligence: A relation mediated by executive functions. Music Percept Interdiscip J. 2011;29: 195–201. doi: 10.1525/mp.2011.29.2.195

[62] Schellenberg EG. Long-term positive associations between music lessons and IQ. J Educ Psychol. 2006;98: 457. doi: 10.1037/0022-0663.98.2.457

[63] Schellenberg EG. Examining the association between music lessons and intelligence. Br J Psychol. 2011;102: 283–302. doi: 10.1111/j.2044-8295.2010.02000.x 21751987

[64] Cooper PK. It’s all in your head: A meta-analysis on the effects of music training on cognitive measures in schoolchildren. Int J Music Educ. 2020;38: 321–336. doi: 10.1177/0255761419881495

[65] Theorell T, Ullén F. Epidemiological studies of the relationship between cultural experiences and public health. Oxf Textb Creat Arts Health Wellbeing Int Perspect Pract Policy Res. 2016; 55–72.

[66] AudienceNet. Multi-Channel Music Research 2015 (a study prepared fro BPI and ERA). 2015. https://www.statista.com/statistics/539816/hours-adults-spent-per-day-listening-to-music-in-the-uk-by-gender/.

[67] Gracie C, Sinha R. Gracie Management Music Consumption Model™ Report. 2012. http://www.graciemgt.com/wp-content/uploads/2012/09/Gracie-Management-Music-Consumption-Model%E2%84%A2-Report.pdf.

[68] Montoro-Pons JD, Cuadrado-García M. Live and prerecorded popular music consumption. J Cult Econ. 2011;35: 19–48. doi: 10.1007/s10824-010-9130-2

[69] Rasmusson X, Fowler A. Older adults use of music in daily life: potential for self-administered therapy. Int J Multidiscip Acad Res. 2016;4: 77–84.

[70] Calvin CM, Deary IJ, Fenton C, Roberts BA, Der G, Leckenby N, et al. Intelligence in youth and all-cause-mortality: systematic review with meta-analysis. Int J Epidemiol. 2010;40: 626–644. doi: 10.1093/ije/dyq190 21037248 PMC3147066

[71] Davey Smith G, Gunnell D, Ben-Shlomo Y. Life-course approaches to socio-economic differentials in cause-specific adult mortality. Poverty Inequal Health Int Perspect Oxf Univ Press N Y. 2001.

[72] Fors S, Lennartsson C, Lundberg O. Childhood living conditions, socioeconomic position in adulthood, and cognition in later life: exploring the associations. J Gerontol B Psychol Sci Soc Sci. 2009;64: 750–757. doi: 10.1093/geronb/gbp029 19420323

[73] Leopold L, Engelhartdt H. Education and physical health trajectories in old age. Evidence from the Survey of Health, Ageing and Retirement in Europe (SHARE). Int J Public Health. 2013;58: 23–31. doi: 10.1007/s00038-012-0399-0 22918517

[74] Luchetti M, Terracciano A, Stephan Y, Sutin AR. Personality and cognitive decline in older adults: Data from a longitudinal sample and meta-analysis. J Gerontol B Psychol Sci Soc Sci. 2016;71: 591–601. doi: 10.1093/geronb/gbu184 25583598 PMC4903032

[75] Njegovan V, Man-Son-Hing M, Mitchell SL, Molnar FJ. The hierarchy of functional loss associated with cognitive decline in older persons. J Gerontol A Biol Sci Med Sci. 2001;56: M638–M643. doi: 10.1093/gerona/56.10.m638 11584037

[76] Hambrick DZ, Tucker-Drob EM. The genetics of music accomplishment: Evidence for gene–environment correlation and interaction. Psychon Bull Rev. 2015;22: 112–120. doi: 10.3758/s13423-014-0671-9 24957535

[77] Mosing MA, Madison G, Pedersen NL, Ullén F. Investigating cognitive transfer within the framework of music practice: Genetic pleiotropy rather than causality. Dev Sci. 2016;19: 504–512. doi: 10.1111/desc.12306 25939545

[78] LeBlanc A, Jin YC, Stamou L, McCrary J. Effect of age, country, and gender on music listening preferences. Bull Counc Res Music Educ. 1999; 72–76.

[79] MacDonald R, Hargreaves DJ, Miell D. Handbook of musical identities. Oxford University Press; 2017.

[80] Colby SM, Clark MA, Rogers ML, Ramsey S, Graham AL, Boergers J, et al. Development and reliability of the lifetime interview on smoking trajectories. Nicotine Tob Res. 2011;14: 290–298. doi: 10.1093/ntr/ntr212 21994340 PMC3281239

[81] Vuillemin A, Guillemin F, Denis G, Huot J, Jeandel C. A computer-assisted assessment of lifetime physical activity: reliability and validity of the QUANTAP software. Rev Epidemiol Sante Publique. 2000;48: 157–167. 10804425

[82] Roberts RO, Geda YE, Knopman DS, Cha RH, Pankratz VS, Boeve BF, et al. The Mayo Clinic Study of Aging: design and sampling, participation, baseline measures and sample characteristics. Neuroepidemiology. 2008;30: 58–69. doi: 10.1159/000115751 18259084 PMC2821441

[83] Huisman M, Poppelaars J, van der Horst M, Beekman AT, Brug J, Van Tilburg TG, et al. Cohort profile: the longitudinal aging study Amsterdam. Int J Epidemiol. 2011;40: 868–876. doi: 10.1093/ije/dyq219 21216744

[84] Lagergren M, Fratiglioni L, Hallberg IR, Berglund J, Elmståhl S, Hagberg B, et al. A longitudinal study integrating population, care and social services data. The Swedish National study on Aging and Care (SNAC). Aging Clin Exp Res. 2004;16: 158–168. doi: 10.1007/BF03324546 15195992

[85] Pitts S. Valuing musical participation. Routledge; 2016.

[86] Pitts SE, Robinson K. Dropping in and dropping out: experiences of sustaining and ceasing amateur participation in classical music. Br J Music Educ. 2016;33: 327–346. doi: 10.1017/S0265051716000152

[87] Turton A, Durrant C. A study of adults’ attitudes, perceptions and reflections on their singing experience in secondary school: some implications for music education. Br J Music Educ. 2002;19: 31–48. doi: 10.1017/S0265051702000128

[88] Ruddock E, Leong S. ‘I am unmusical!’: The verdict of self-judgement. Int J Music Educ. 2005;23: 9–22. doi: 10.1177/0255761405050927

