# Supplementary material for: The Edinburgh Lifetime Musical Experience Questionnaire (ELMEQ): Responses and non-musical correlates in the Lothian Birth Cohort 1936
Source: PLoS One. 2021 Jul 15;16(7):e0254176. doi: 10.1371/journal.pone.0254176 (PMC8282069; doi:10.1371/journal.pone.0254176)

**S1 Fig. Flowchart showing the number of participants excluded from the analytical sample for the factor analysis and structural equation models.**


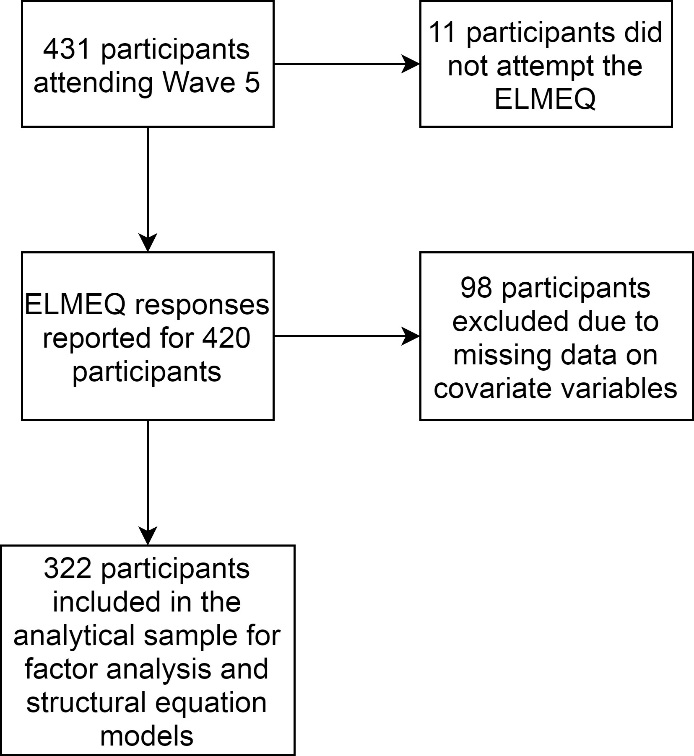

Supplement: S1 Fig — (DOCX) [file pone.0254176.s001.docx]
