# Supplementary material for: The Edinburgh Lifetime Musical Experience Questionnaire (ELMEQ): Responses and non-musical correlates in the Lothian Birth Cohort 1936
Source: PLoS One. 2021 Jul 15;16(7):e0254176. doi: 10.1371/journal.pone.0254176 (PMC8282069; doi:10.1371/journal.pone.0254176)
Supplement: S2 Fig — (DOCX) [file pone.0254176.s002.docx]

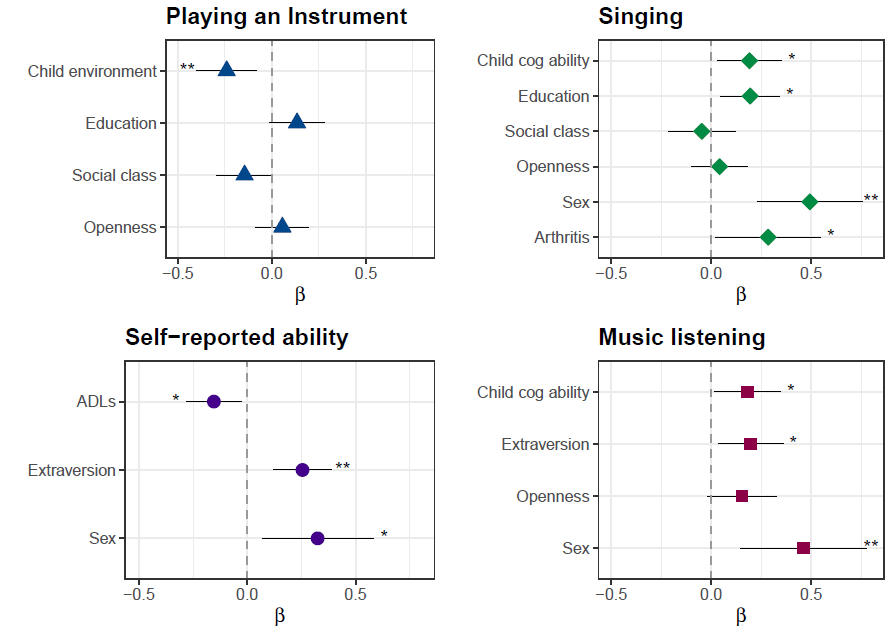
**S2 Fig. Non-musical variables associated with the musical experience domains.**

Figure shows standardised estimates and 95% confidence intervals from the final model which includes variables that were statistically significantly related to musical experience domains in models that separately tested for associations with childhood, older-age, and personality variables.

Sex coded as 0 = male, 1 = female. History of arthritis is coded as 0 = no, 1 = yes. For childhood environment, lower scores indicate less environmental deprivation. Lower scores on social class indicate a more professional occupation. Lower scores on the activities of daily living (ADLs) scale indicate fewer constraints. ‘Openness’ = personality trait openness to experience.

***p* <.001, **p* <.05.
