# Supplementary material for: The Edinburgh Lifetime Musical Experience Questionnaire (ELMEQ): Responses and non-musical correlates in the Lothian Birth Cohort 1936
Source: PLoS One. 2021 Jul 15;16(7):e0254176. doi: 10.1371/journal.pone.0254176 (PMC8282069; doi:10.1371/journal.pone.0254176)
Supplement: S3 Fig — (DOCX) [file pone.0254176.s003.docx]

**S3 Fig. Diagram of the final model of lifetime musical experience and its lifespan correlates.**


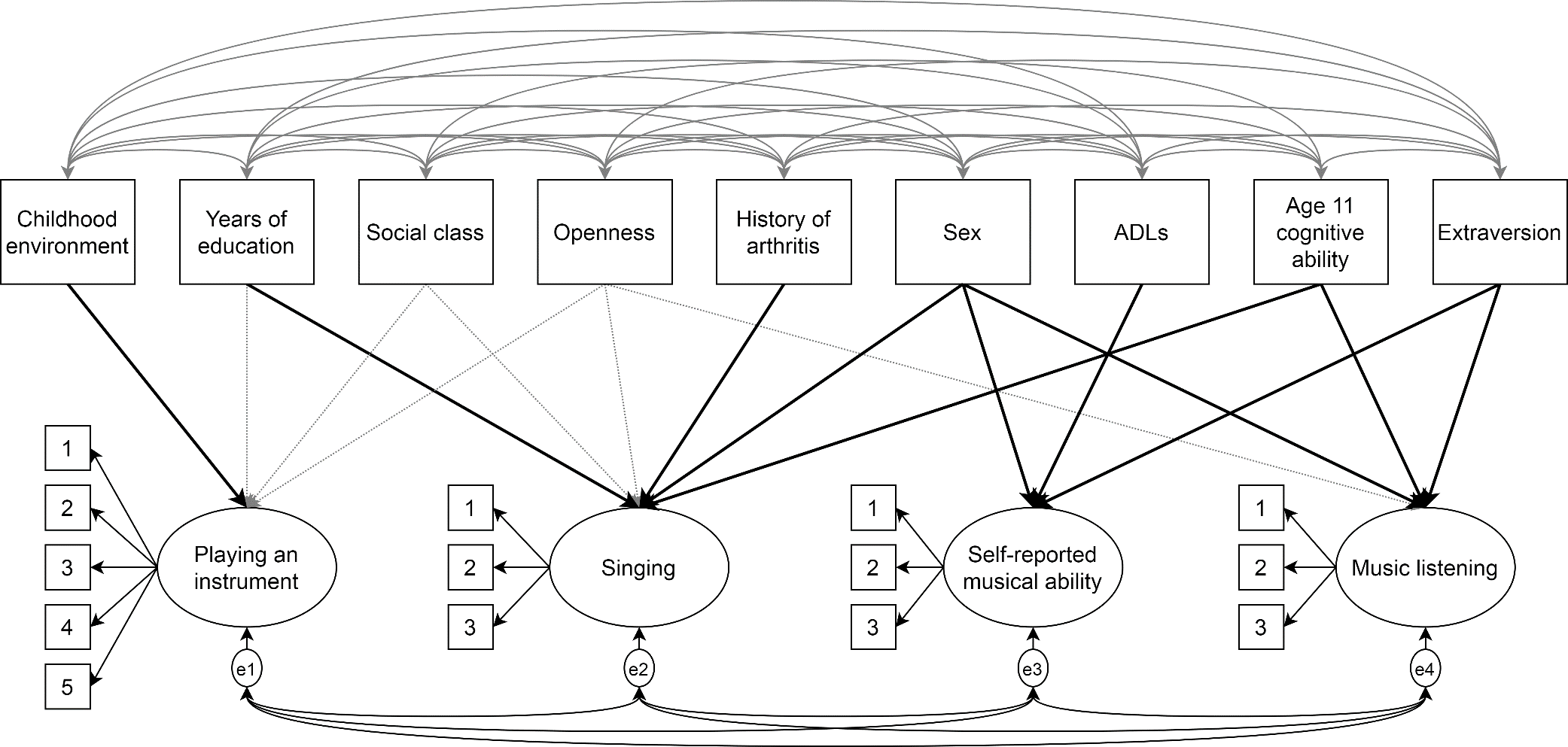


Ellipses represent latent variables, rectangles observed variables, single headed arrows regression paths or factor loadings, and double headed arrows covariances. Bold paths from the covariate variables to the musical experience latent variables indicate statistically significant associations.

Openness = personality trait openness to experience. ADLs = activities of daily living.
