# Supplementary material for: The Edinburgh Lifetime Musical Experience Questionnaire (ELMEQ): Responses and non-musical correlates in the Lothian Birth Cohort 1936
Source: PLoS One. 2021 Jul 15;16(7):e0254176. doi: 10.1371/journal.pone.0254176 (PMC8282069; doi:10.1371/journal.pone.0254176)
Supplement: S1 Table — (DOCX) [file pone.0254176.s004.docx]

| **S1 Table.** **Categories of musical experience used in observational studies of cognitive ageing or dementia risk.** | | |
| --- | --- | --- |
| Study | Type of assessment | Categories of musical experience used in analysis |
| Verghese et al. (2003) | Leisure activities interview: frequency of playing | **Plays musical instrument frequently** (several days or more per week)  **Plays musical instrument rarely/never** (weekly or less) |
| Grant & Brody (2004) | Interview: age at training onset, absolute pitch, current playing | Former members of an orchestra only |
| Hughes et al. (2010) | Leisure activities questionnaire: whether plays a musical instrument | **Plays musical instrument** (yes/no) |
| Hanna-Pladdy & MacKay (2011) | Questionnaire (items not reported) | **High activity musician** (at least 10 years’ experience playing).  **Low activity musician** (1-9 years’ experience playing)  **Non-musician** (cannot play an instrument)  Also examined current/past musical activity, years and frequency of activity, instrument and training and type |
| Hanna-Pladdy & Gajewski (2012) | Structured interview: years of formal training, age of acquisition, practice routines | **Musician** (more than 10 years of musical participation)  **Non-musician** (less than 1 year of musical participation)  Also examined age of acquisition |
| Amer et al. (2013) | Questionnaire (items not reported) | **Professional musician** (instrumentalist or vocalist with extensive formal training)  **Non-musician** (minimal/no music education outside of school and no history of playing a musical instrument or singing regularly) |
| Balbag et al. (2014) | Leisure activities questionnaire: playing new/familiar music, frequency playing, whether currently plays, age stopped playing | **Musician** (plays new and or familiar music frequently or occasionally at time of assessment or stopped playing within 5 years prior to dementia onset)  **Non-musician** (does not play music at time of assessment or within 5 years of dementia onset) |
| Fauvel et al. (2014) | Questionnaire: age at training onset, frequency of training | **Amateur musician** (recruited from French conservatory/music school; at least 4 years of uninterrupted study)  **Non-musician** (recruited from the general population) |
| Gooding et al. (2014) | Questionnaire: musical notation, whether currently playing or singing, which instruments, age at training onset, years played, frequency playing/singing, proficiency, enjoyment playing/listening to music | **High musical knowledge** (based on a music theory test)  **Medium musical knowledge** (based on a music theory test)  **Low musical knowledge** (based on a music theory test)  Also compared groups who could and could not read musical notation |
| Moussard et al. (2016) | Phone interview: age at training onset, years played, frequency playing/singing, type of instrument, type of training, qualifications/teaching | **Musician** (professional/amateur with advanced musical experience)  **Non-musician** (minimal/no music education outside of school, no history of playing a musical instrument or singing regularly) |
| Mansens et al. (2017) | Questionnaire: whether plays/sings, which instrument, frequency of playing/singing per two weeks, minutes of practice | **Makes music** (plays an instrument/sings)  **Does not make music** (does not play an instrument/sing)  **Time spent making music per average two weeks** |
| Gray & Gow (2019) | Questionnaire (items not reported) | **Musician** (plays/played a musical instrument)  **Non-Musician** (never played an instrument or stopped within a year) |
| Strong & Midden (2020) | Questionnaire: age at training onset, instruments played, ensemble involvement, years of private lessons, average number of hours played per week, ability to compose or improvise. | **Musician** (currently plays and has had lessons for more than one year)  **Former musician** (no longer plays and has had lessons for more than one year)  **Non-musician** (less than one year of lessons) |

**References**

Amer, T., Kalender, B., Hasher, L., Trehub, S. E., & Wong, Y. (2013). Do older professional musicians have cognitive advantages? *PloS One*, *8*(8), e71630. https://doi.org/10.1371/journal.pone.0071630

Balbag, M. A., Pedersen, N. L., & Gatz, M. (2014). Playing a musical instrument as a protective factor against dementia and cognitive impairment: A population-based twin study. *International Journal of Alzheimer’s Disease*, *2014*. https://doi.org/10.1155/2014/836748

Fauvel, B., Groussard, M., Mutlu, J., Arenaza-Urquijo, E. M., Eustache, F., Desgranges, B., & Platel, H. (2014). Musical practice and cognitive aging: Two cross-sectional studies point to phonemic fluency as a potential candidate for a use-dependent adaptation. *Frontiers in Aging Neuroscience*, *6*, 227. https://doi.org/10.3389/fnagi.2014.00227

Gooding, L. F., Abner, E. L., Jicha, G. A., Kryscio, R. J., & Schmitt, F. A. (2014). Musical training and late-life cognition. *American Journal of Alzheimer’s Disease & Other Dementias*, *29*(4), 333–343. https://doi.org/10.1177/1533317513517048

Grant, M. D., & Brody, J. A. (2004). Musical experience and dementia. Hypothesis. *Aging Clinical and Experimental Research*, *16*(5), 403–405. https://doi.org/10.1007/BF03324571

Gray, R., & Gow, A. J. (2019). How is musical activity associated with cognitive ability in later life? *Aging, Neuropsychology, and Cognition*, 1–19. https://doi.org/10.1080/13825585.2019.1660300

Hanna-Pladdy, B., & Gajewski, B. (2012). Recent and past musical activity predicts cognitive aging variability: Direct comparison with general lifestyle activities. *Frontiers in Human Neuroscience*, *6*, 198. https://doi.org/10.3389/fnhum.2012.00198

Hanna-Pladdy, B., & MacKay, A. (2011). The relation between instrumental musical activity and cognitive aging. *Neuropsychology*, *25*(3), 378. https://doi.org/10.1037/a0021895

Hughes, T. F., Chang, C.-C. H., Vander Bilt, J., & Ganguli, M. (2010). Engagement in reading and hobbies and risk of incident dementia: The MoVIES project. *American Journal of Alzheimer’s Disease & Other Dementias*, *25*(5), 432–438. https://doi.org/10.1177/1533317510368399

Mansens, D., Deeg, D. J. H., & Comijs, H. C. (2017). The association between singing and/or playing a musical instrument and cognitive functions in older adults. *Aging & Mental Health*, 1–8. https://doi.org/10.1080/13607863.2017.1328481

Moussard, A., Bermudez, P., Alain, C., Tays, W., & Moreno, S. (2016). Life-long music practice and executive control in older adults: An event-related potential study. *Brain Research*, *1642*, 146–153. https://doi.org/10.1016/j.brainres.2016.03.028

Strong, J. V., & Midden, A. (2020). Cognitive differences between older adult instrumental musicians: Benefits of continuing to play. *Psychology of Music*, *48*(1), 67–83. https://doi.org/10.1177/0305735618785020

Verghese, J., Lipton, R. B., Katz, M. J., Hall, C. B., Derby, C. A., Kuslansky, G., Ambrose, A. F., Sliwinski, M., & Buschke, H. (2003). Leisure activities and the risk of dementia in the elderly. *New England Journal of Medicine*, *348*(25), 2508–2516. https://doi.org/10.1056/NEJMoa022252
