# Supplementary material for: The Edinburgh Lifetime Musical Experience Questionnaire (ELMEQ): Responses and non-musical correlates in the Lothian Birth Cohort 1936
Source: PLoS One. 2021 Jul 15;16(7):e0254176. doi: 10.1371/journal.pone.0254176 (PMC8282069; doi:10.1371/journal.pone.0254176)
Supplement: S2 Table — (DOCX) [file pone.0254176.s005.docx]

**S2 Table.** **Characteristics (assessed at Wave 1) of participants who responded to the ELMEQ (N=420) and of those who did not (N=671).**

| Variable | Leavers/non-responders (N=671^1^) | Wave 5 Responders (N=420) | Total (N=1,091) | *p* |
| --- | --- | --- | --- | --- |
| Sex |  |  |  | 0.386^2^ |
| - Female (%) | 327 (48.7%) | 216 (51.4%) | 543 (49.8%) |  |
| Age 11 cognitive ability |  |  |  | < 0.001^3^ |
| - Mean (SD) | 98.29 (14.95) | 102.75 (14.67) | 100.00 (14.99) |  |
| Childhood environment |  |  |  | 0.014^3^ |
| - Mean (SD) | 0.14 (2.49) | -0.23 (2.26) | 0.00 (2.41) |  |
| Years of education |  |  |  | < 0.001^3^ |
| - Mean (SD) | 10.64 (1.09) | 10.91 (1.18) | 10.741 (1.13) |  |
| Father’s social class |  |  |  | 0.093^3^ |
| - Mean (SD) | 2.951 (0.93) | 2.847 (0.96) | 2.909 (0.94) |  |
| Adult social class |  |  |  | < 0.001^3^ |
| - Mean (SD) | 2.523 (0.90) | 2.214 (0.91) | 2.404 (0.91) |  |
| Environmental quality |  |  |  | < 0.001^3^ |
| - Mean (SD) | 5.98 (2.19) | 6.69 (1.85) | 6.26 (2.09) |  |

^1^Participants did not respond to the ELMEQ either due to leaving the study before Wave 5 (N=660) or not responding to the ELMEQ at Wave 5 (N=11). These groups were combined as the number of participants who did not respond to the ELMEQ at Wave 5 was too low for statistical comparison. ^2^Pearson’s Chi-squared test.

^3^Linear Model ANOVA.

Lower scores on childhood environment indicate a lower level of deprivation. Lower scores on father’s social class and adult social class indicate a more professional occupation. Higher scores on environmental quality indicate better quality. Lower scores on the activities of daily living scale indicate fewer constraints.
