# Supplementary material for: The Edinburgh Lifetime Musical Experience Questionnaire (ELMEQ): Responses and non-musical correlates in the Lothian Birth Cohort 1936
Source: PLoS One. 2021 Jul 15;16(7):e0254176. doi: 10.1371/journal.pone.0254176 (PMC8282069; doi:10.1371/journal.pone.0254176)
Supplement: S3 Table — (DOCX) [file pone.0254176.s006.docx]

**S3 Table.** **Characteristics** **of participants who responded to the ELMEQ and number of participants with missing data on the non-musical covariate variables.**

| Variable | Overall (N=420) | Missing N |
| --- | --- | --- |
| Sex |  |  |
| - Female (%) | 216 (51.4%) | 0 |
| Age 11 cognitive ability |  |  |
| - Mean (SD) | 102.75 (14.67) | 26 |
| Childhood environment |  |  |
| - Mean (SD) | -0.23 (2.26) | 1 |
| Years of education |  |  |
| - Mean (SD) | 10.91 (1.18) | 0 |
| Father’s social class |  |  |
| - Mean (SD) | 2.85 (0.96) | 34 |
| Participant’s adult social class |  |  |
| - Mean (SD) | 2.21 (0.91) | 7 |
| Environmental quality |  |  |
| - Mean (SD) | 6.69 (1.85) | 2 |
| Activities of daily living |  |  |
| - Mean (SD) | 1.88 (2.97) | 0 |
| History of diabetes |  |  |
| - Yes (%) | 50 (11.9%) | 0 |
| History of CVD |  |  |
| - Yes (%) | 165 (39.5%) | 2 |
| History of stroke |  |  |
| - Yes (%) | 57 (13.7%) | 3 |
| History of cancer |  |  |
| - Yes (%) | 94 (22.4%) | 1 |
| History of Parkinson’s |  |  |
| - Yes (%) | 6 (1.4%) | 0 |
| History of dementia |  |  |
| - Yes (%) | 11 (2.6%) | 0 |
| History of arthritis |  |  |
| - Yes (%) | 213 (51.6%) | 7 |
| Extraversion |  |  |
| - Mean (SD) | 21.51 (7.29) | 16 |
| Agreeableness |  |  |
| - Mean (SD) | 30.90 (5.28) | 11 |
| Conscientiousness |  |  |
| - Mean (SD) | 27.50 (6.04) | 13 |
| Emotional stability |  |  |
| - Mean (SD) | 25.73 (6.89) | 14 |
| Openness to experience |  |  |
| - Mean (SD) | 23.61 (5.91) | 14 |

Lower scores on childhood environment indicate a lower level of deprivation. Lower scores on father’s social class and adult social class indicate a more professional occupation. Higher scores on environmental quality indicate better quality. Lower scores on the activities of daily living scale indicate fewer constraints.
