# Supplementary material for: The Edinburgh Lifetime Musical Experience Questionnaire (ELMEQ): Responses and non-musical correlates in the Lothian Birth Cohort 1936
Source: PLoS One. 2021 Jul 15;16(7):e0254176. doi: 10.1371/journal.pone.0254176 (PMC8282069; doi:10.1371/journal.pone.0254176)
Supplement: S4 Table — (DOCX) [file pone.0254176.s007.docx]

**S4 Table.** **Characteristics of participants included and excluded from the analytical sample due to missing data points.**

| Variable | Excluded  (N=98) | Included  (N=322) | Total  (N=420) | *p* |
| --- | --- | --- | --- | --- |
| Played an instrument |  |  |  | 0.979^1^ |
| - Yes (%) | 37 (40.2%) | 130 (40.4%) | 167 (40.3%) |  |
| Ever sung |  |  |  | 0.026^1^ |
| - Yes (%) | 26 (28.0%) | 131 (40.7%) | 157 (37.8%) |  |
| Sex |  |  |  | 0.747^1^ |
| - Female (%) | 49 (50.0%) | 167 (51.9%) | 216 (51.4%) |  |
| Age 11 cognitive ability |  |  |  | 0.133^2^ |
| - Mean (SD) | 100.40 (16.13) | 103.28 (14.30) | 102.75 (14.67) |  |
| Childhood environment |  |  |  | 0.403^2^ |
| - Mean (SD) | -0.058 (2.70) | -0.278 (2.12) | -0.227 (2.26) |  |
| Years of education |  |  |  | 0.687^2^ |
| - Mean (SD) | 10.867 (1.25) | 10.922 (1.162) | 10.910 (1.18) |  |
| Father’s social class |  |  |  | 0.456^2^ |
| - Mean (SD) | 2.77 (1.00) | 2.87 (0.95) | 2.85 (0.96) |  |
| Participant’s social class |  |  |  | 0.295^2^ |
| - Mean (SD) | 2.30 (0.98) | 2.19 (0.88) | 2.21 (0.91) |  |
| Environmental quality |  |  |  | < 0.001^2^ |
| - Mean (SD) | 6.10 (2.15) | 6.87 (1.71) | 6.69 (1.85) |  |
| Activities of daily living |  |  |  | 0.819^2^ |
| - Mean (SD) | 1.94 (2.97) | 1.86 (2.98) | 1.88 (2.97) |  |
| History of diabetes |  |  |  | 0.905^1^ |
| - Yes (%) | 12 (12.2%) | 38 (11.8%) | 50 (11.9%) |  |
| History of CVD |  |  |  | 0.146^1^ |
| - Yes (%) | 44 (45.8%) | 121 (37.6%) | 165 (39.5%) |  |
| History of stroke |  |  |  | 0.306^1^ |
| - Yes (%) | 16 (16.8%) | 41 (12.7%) | 57 (13.7%) |  |
| History of cancer |  |  |  | 0.534^1^ |
| - Yes (%) | 24 (24.7%) | 70 (21.7%) | 94 (22.4%) |  |
| History of Parkinson’s |  |  |  | 0.560^1^ |
| - Yes (%) | 2 (2.0%) | 4 (1.2%) | 6 (1.4%) |  |
| History of dementia |  |  |  | 0.258^1^ |
| - Yes (%) | 1 (1.0%) | 10 (3.1%) | 11 (2.6%) |  |
| History of arthritis |  |  |  | 0.825^1^ |
| - Yes (%) | 46 (50.5%) | 167 (51.9%) | 213 (51.6%) |  |
| Extraversion |  |  |  | 0.903^2^ |
| - Mean (SD) | 21.60 (7.22) | 21.49 (7.32) | 21.51 (7.29) |  |
| Agreeableness |  |  |  | 0.776^2^ |
| - Mean (SD) | 30.76 (5.18) | 30.94 (5.32) | 30.90 (5.28) |  |
| Conscientiousness |  |  |  | 0.634^2^ |
| - Mean (SD) | 27.22 (5.55) | 27.58 (6.17) | 27.50 (6.04) |  |
| Emotional stability |  |  |  | 0.809^2^ |
| - Mean (SD) | 25.57 (7.06) | 25.78 (6.86) | 25.73 (6.89) |  |
| Openness to experience |  |  |  | 0.877^2^ |
| - Mean (SD) | 23.70 (5.59) | - 1. (6.00) | 23.613 (5.91) |  |

^1^Pearson’s Chi-squared test.

^2^Linear Model ANOVA.

Lower scores on childhood environment indicate a lower level of deprivation. Lower scores on father’s social class and adult social class indicate a more professional occupation. Higher scores on environmental quality indicate better quality. Lower scores on the activities of daily living scale indicate fewer constraints.
