# Supplementary material for: The Edinburgh Lifetime Musical Experience Questionnaire (ELMEQ): Responses and non-musical correlates in the Lothian Birth Cohort 1936
Source: PLoS One. 2021 Jul 15;16(7):e0254176. doi: 10.1371/journal.pone.0254176 (PMC8282069; doi:10.1371/journal.pone.0254176)
Supplement: S5 Table — (DOCX) [file pone.0254176.s008.docx]

| **S5 Table.** **Responses to Section 1: Experience Playing a Music Instrument.** | | |
| --- | --- | --- |
| Item | N of Responses  (% of total N) | Missing/NA |
| On how many instruments could/can you perform a short piece of music |  | 4 |
| - 0 | 11 (6.7%) |  |
| - 1 | 115 (70.6%) |  |
| - 2 | 29 (17.8%) |  |
| - 3 | 8 (4.9%) |  |
| Type of instrument (1^st^ instrument) |  | 1 |
| - Piano | 112 (67.5%) |  |
| - Other (see Sup Table 5.1) | 54 (32.5%) |  |
| Age began playing |  | 1 |
| - 4-5 | 5 (3.0%) |  |
| - 6-7 | 33 (19.9%) |  |
| - 8-11 | 71 (42.8%) |  |
| - 12 or older | 57 (34.3%) |  |
| Formal instrumental training |  | 1 |
| - Yes | 143 (86.1%) |  |
| Years of formal instrumental training^1^ |  | 1/23 |
| - 0-1 | 14 (9.8%) |  |
| - 2-5 | 83 (58.0%) |  |
| - 6-10 | 39 (27.3%) |  |
| - 11-20 | 6 (4.2%) |  |
| - 21+ | 1 (0.7%) |  |
| Years of regular practice |  | 14 |
| - 0-5 | 70 (45.8%) |  |
| - 6-10 | 29 (19.0%) |  |
| - 11-20 | 15 (9.8%) |  |
| - 21-40 | 14 (9.2%) |  |
| - 41+ | 25 (16.3%) |  |
| Hours of practice per week (during period of regular practice) |  | 13 |
| - 0-1 | 48 (31.2%) |  |
| - 2-3 | 59 (38.3%) |  |
| - 4-6 | 36 (23.4%) |  |
| - 7-13 | 7 (4.5%) |  |
| - 14+ | 4 (2.6%) |  |
| Level of performance |  | 15 |
| - Beginner | 61 (40.1%) |  |
| - Intermediate | 76 (50.0%) |  |
| - Advanced | 9 (5.9%) |  |
| - Semi-professional | 2 (1.3%) |  |
| - Professional | 4 (2.6%) |  |
| Regularly read from musical score |  | 11 |
| - Yes | 133 (85.3%) |  |
| Regularly played pieces by ear |  | 20 |
| - Yes | 49 (33.3%) |  |
| Regularly improvised |  | 24 |
| - Yes | 32 (22.4%) |  |
| Currently playing |  | 1 |
| - Yes | 39 (23.5%) |  |
| Hours currently playing per week^2^ |  | 1/127 |
| - 0-1 | 21 (53.8%) |  |
| - 2-3 | 12 (30.8%) |  |
| - 4-6 | 3 (7.7%) |  |
| - 7-13 | 3 (7.7%) |  |

Showing responses only for participants who responded “Yes” to item 1 (Have you ever learned to play a musical instrument?) N = 167. Percentage is based on the number of participants who responded to that question. The last column shows the number of missing responses and the number of participants who did not respond because the question did not apply (NA).

^1^5 participants who reported no formal training responded to this question; their responses were recoded as not applicable.

^2^16 participants who reported not currently playing responded to this question, their responses were recoded as not applicable.

| **S5.1 Table. Additional Responses to Section 1: Type of Musical Instrument.** | | |
| --- | --- | --- |
|  | N | % of total (N= 166) |
| Piano | 112 | 67.5% |
| Bagpipes | 9 | 5.4% |
| Violin | 9 | 5.4% |
| Accordion | 7 | 4.2% |
| Guitar | 5 | 3.0% |
| Keyboard | 4 | 2.4% |
| Clarinet | 3 | 1.8% |
| Piano organ | 3 | 1.8% |
| Recorder | 3 | 1.8% |
| Drums | 2 | 1.2% |
| Trumpet | 2 | 1.2% |
| Banjo | 1 | 0.6% |
| Cello | 1 | 0.6% |
| Cornet | 1 | 0.6% |
| Flute | 1 | 0.6% |
| Mouth organ | 1 | 0.6% |
| Tenor horn | 1 | 0.6% |
| Ukulele | 1 | 0.6% |
