# Supplementary material for: The Edinburgh Lifetime Musical Experience Questionnaire (ELMEQ): Responses and non-musical correlates in the Lothian Birth Cohort 1936
Source: PLoS One. 2021 Jul 15;16(7):e0254176. doi: 10.1371/journal.pone.0254176 (PMC8282069; doi:10.1371/journal.pone.0254176)
Supplement: S6 Table — (DOCX) [file pone.0254176.s009.docx]

| **S6 Table.** **Responses to Section 1: Experience Playing Multiple Instruments.** | | |
| --- | --- | --- |
|  | N of Responses  (% of total N) | Missing/NA |
| Can/could perform a short piece on at least two instruments |  | 4 |
| - Yes | 37 (22.7%) |  |
| Age began 2nd instrument^1^ |  | 6/126 |
| - Mean (SD) | 23.600 (19.176) |  |
| Years played 2nd instrument^1^ |  | 7/126 |
| - Mean (SD) | 15.442 (22.842) |  |
| Can/could perform a short piece on at least three instruments |  | 4 |
| - Yes | 8 (4.9%) |  |
| Age began 3rd instrument^2^ |  | 4/155 |
| - Mean (SD) | 38.188 (20.185) |  |
| Years played 3rd instrument^2^ |  | 4/155 |
| - Mean (SD) | 4.406 (2.835) |  |

Showing responses only for participants who responded “Yes” to item 1 (Have you ever learned to play a musical instrument?), N = 167. Percentage is based on the number of participants who responded to that question. The last column shows the number of missing responses and the number of participants who did not respond because the question did not apply.

^1^2 participants who reported playing fewer than two instruments responded to this question, their responses were recoded as not applicable.

^2^1 participant who reported playing fewer than three instruments responded to this question, their response was recoded as not applicable.

NA = not applicable*.*
