# Supplementary material for: The Edinburgh Lifetime Musical Experience Questionnaire (ELMEQ): Responses and non-musical correlates in the Lothian Birth Cohort 1936
Source: PLoS One. 2021 Jul 15;16(7):e0254176. doi: 10.1371/journal.pone.0254176 (PMC8282069; doi:10.1371/journal.pone.0254176)
Supplement: S7 Table — (DOCX) [file pone.0254176.s010.docx]

| **S7 Table.** **Responses to Section 1: Experience Playing in a Band or Ensemble.** | | |
| --- | --- | --- |
|  | N of Responses  (% of total N) | Missing/NA |
| Ever played in a group or band |  | 8 |
| - Yes | 30 (18.9%) |  |
| Years played with a group or band^1^ |  | 9/129 |
| - 0-5 | 14 (48.3%) |  |
| - 6-10 | 7 (24.1%) |  |
| - 11-20 | 3 (10.3%) |  |
| - 21-40 | 1 (3.4%) |  |
| - 41+ | 4 (13.8%) |  |
| Hours of practice per week with a group or band^1^ |  | 9/129 |
| - 0-1 | 5 (17.2%) |  |
| - 2-3 | 16 (55.2%) |  |
| - 4-6 | 7 (24.1%) |  |
| - 14+ | 1 (3.4%) |  |

Showing responses only for participants who responded “Yes” to item 1 (Have you ever learned to play a musical instrument?). Percentage is based on the number of participants who responded to that question. The last column shows the number of missing responses and the number of participants who did not respond because the question did not apply (NA).

^1^Two participants who reported not playing in a band or ensemble responded to this question, their responses were recoded as not applicable.
