# Supplementary material for: The Edinburgh Lifetime Musical Experience Questionnaire (ELMEQ): Responses and non-musical correlates in the Lothian Birth Cohort 1936
Source: PLoS One. 2021 Jul 15;16(7):e0254176. doi: 10.1371/journal.pone.0254176 (PMC8282069; doi:10.1371/journal.pone.0254176)
Supplement: S8 Table — (DOCX) [file pone.0254176.s011.docx]

| **S8 Table.** **Responses to Section 2: Experience Singing.** | | |
| --- | --- | --- |
|  | N of Responses  (% of total N) | Missing/NA |
| Age began singing |  | 1 |
| - 4-5 | 1 (0.6%) |  |
| - 6-7 | 13 (8.3%) |  |
| - 8-11 | 39 (25.0%) |  |
| - 12+ | 103 (66.0%) |  |
| Years of singing |  | 2 |
| - 0-4 | 69 (44.5%) |  |
| - 5-10 | 45 (29.0%) |  |
| - 11-12 | 15 (9.7%) |  |
| - 21-40 | 12 (7.7%) |  |
| - 41+ | 14 (9.0%) |  |
| Hours of practice per week |  | 2 |
| - 0-1 | 67 (43.2%) |  |
| - 2-3 | 76 (49.0%) |  |
| - 4-6 | 10 (6.5%) |  |
| - 7-13 | 2 (1.3%) |  |
| Any solo vocal training |  | 1 |
| - Yes | 16 (10.3%) |  |
| Years of solo vocal training |  | 2/140 |
| - 2-5 | 12 (80.0%) |  |
| - 6-10 | 2 (13.3%) |  |
| - 11-20 | 1 (6.7%) |  |

Showing responses only for participants who responded “Yes” to item 1 (Have you ever sung in a group?), N = 157. Percentage is based on the number of participants who responded to that question. The last column shows the number of missing responses and the number of participants who did not respond because the question did not apply (NA).
