# Supplementary material for: The Edinburgh Lifetime Musical Experience Questionnaire (ELMEQ): Responses and non-musical correlates in the Lothian Birth Cohort 1936
Source: PLoS One. 2021 Jul 15;16(7):e0254176. doi: 10.1371/journal.pone.0254176 (PMC8282069; doi:10.1371/journal.pone.0254176)
Supplement: S9 Table — (DOCX) [file pone.0254176.s012.docx]

| **S9 Table.** **Responses to section 3: Reading Music Notation.** | | |
| --- | --- | --- |
|  | N of Responses  (% of total N) | Missing |
| Can read treble clef |  | 9 |
| - Yes | 97 (89.0%) |  |
| Can read bass clef |  | 9 |
| - Yes | 77 (70.6%) |  |
| Can read alto clef |  | 9 |
| - Yes | 24 (22.0%) |  |
| Can read guitar tab |  | 9 |
| - Yes | 3 (2.8%) |  |
| Can read percussion notation |  | 9 |
| - Yes | 4 (3.7%) |  |
| Can read chord symbols |  | 9 |
| - Yes | 34 (31.2%) |  |
| Highest level of sight-reading |  |  |
| - beginner | 54 (45.8%) |  |
| - intermediate | 50 (42.4%) |  |
| - advanced | 10 (8.5%) |  |
| - semi-professional | 3 (2.5%) |  |
| - professional | 1 (0.8%) |  |

Showing responses only for participants who responded “Yes” to item 1 (Have you ever learned to read any kind of musical notation?), N = 118. Percentage is based on the number of participants who responded to that question. The last column shows the number of missing responses.
