# Supplementary material for: The Edinburgh Lifetime Musical Experience Questionnaire (ELMEQ): Responses and non-musical correlates in the Lothian Birth Cohort 1936
Source: PLoS One. 2021 Jul 15;16(7):e0254176. doi: 10.1371/journal.pone.0254176 (PMC8282069; doi:10.1371/journal.pone.0254176)
Supplement: S10 Table — (DOCX) [file pone.0254176.s013.docx]

| **S10 Table.** **Responses to Section 4: Listening to Music.** | | |
| --- | --- | --- |
|  | N of Responses  (% of total N) | Missing |
| Recorded music hours per week |  | 13 |
| - 0-1 | 80 (19.7%) |  |
| - 2-3 | 150 (36.9%) |  |
| - 4-6 | 84 (20.6%) |  |
| - 7-13 | 49 (12.0%) |  |
| - 14+ | 44 (10.8%) |  |
| Concerts/gigs per year |  | 9 |
| - 0-1 | 172 (41.8%) |  |
| - 2-6 | 156 (38.0%) |  |
| - 7-24 | 61 (14.8%) |  |
| - 25-49 | 12 (2.9%) |  |
| - 50+ | 10 (2.4%) |  |
| Clap hands to music |  | 8 |
| - very difficult | 4 (1.0%) |  |
| - difficult | 18 (4.4%) |  |
| - not sure | 45 (10.9%) |  |
| - easy | 172 (41.7%) |  |
| - very easy | 173 (42.0%) |  |
| Dance to music |  | 10 |
| - very difficult | 13 (3.2%) |  |
| - difficult | 49 (12.0%) |  |
| - not sure | 61 (14.9%) |  |
| - easy | 170 (41.5%) |  |
| - very easy | 117 (28.5%) |  |
| Sing a melody in tune |  | 11 |
| - very difficult | 43 (10.5%) |  |
| - difficult | 60 (14.7%) |  |
| - not sure | 88 (21.5%) |  |
| - easy | 143 (35.0%) |  |
| - very easy | 75 (18.3%) |  |
| Parents sang songs |  | 5 |
| - never | 56 (13.5%) |  |
| - rarely | 99 (23.9%) |  |
| - sometimes | 125 (30.1%) |  |
| - regularly | 106 (25.5%) |  |
| - daily | 29 (7.0%) |  |
| Listening to music important^1^ |  | 4/51^1^ |
| - not at all important | 14 (3.8%) |  |
| - not very important | 44 (12.1%) |  |
| - not sure | 37 (10.1%) |  |
| - quite important | 155 (42.5%) |  |
| - very important | 115 (31.5%) |  |
| Strong emotional response to music^1^ |  | 5/51^1^ |
| - not at all | 10 (2.7%) |  |
| - not really | 64 (17.6%) |  |
| - not sure | 51 (14.0%) |  |
| - quite strong | 165 (45.3%) |  |
| - very strong | 74 (20.3%) |  |

Showing responses for all participants who attempted the questionnaire, N = 420. The last column shows the number of missing responses and the number of participants who did not respond because the question did not apply (NA).

^1^The first 51 participants to complete the ELMEQ were given a different version of the questionnaire that did not include the items “How important has listening to music been to you over the course of your life?” and “Would you say that you have strong emotional responses to music?” This is reflected by the number of NAs for these questions.
