# Supplementary material for: The Edinburgh Lifetime Musical Experience Questionnaire (ELMEQ): Responses and non-musical correlates in the Lothian Birth Cohort 1936
Source: PLoS One. 2021 Jul 15;16(7):e0254176. doi: 10.1371/journal.pone.0254176 (PMC8282069; doi:10.1371/journal.pone.0254176)
Supplement: S11 Table — (DOCX) [file pone.0254176.s014.docx]

| **S11 Table.** **Correlations between indicators of *Playing a Musical Instrument*.** | | | | |
| --- | --- | --- | --- | --- |
| Variable | 1 | 2 | 3 | 4 |
| 1. Number of musical instruments | - |  |  |  |
| 2. Years of formal training | .221** | - |  |  |
| 3. Years of regular practice | .268** | .583** | - |  |
| 4. Hours of practice per week | .186* | .182* | .185* | - |
| 5. Level of performance | .159 | .531** | .522** | .427** |
| Correlations are non-parametric Spearman’s rho. An extra (lowest) category was created for years of formal instrumental training for participants who reported no formal training.  **p* < .05. ***p* < .01. | | | | |
