# Supplementary material for: The Edinburgh Lifetime Musical Experience Questionnaire (ELMEQ): Responses and non-musical correlates in the Lothian Birth Cohort 1936
Source: PLoS One. 2021 Jul 15;16(7):e0254176. doi: 10.1371/journal.pone.0254176 (PMC8282069; doi:10.1371/journal.pone.0254176)
Supplement: S12 Table — (DOCX) [file pone.0254176.s015.docx]

| **S12 Table. Correlations between indicators of *Singing*.** | | |
| --- | --- | --- |
|  | 1 | 2 |
| 1. Years of singing | - |  |
| 2. Hours of practice per week | .207** | - |
| 3. Years of solo vocal training | .127 | .259** |
| Correlations are non-parametric Spearman’s rho. An extra (lowest) category was created for years of solo vocal training for participants who reported no solo training.  **p* < .05. ***p* < .01. | | |
