# Supplementary material for: The Edinburgh Lifetime Musical Experience Questionnaire (ELMEQ): Responses and non-musical correlates in the Lothian Birth Cohort 1936
Source: PLoS One. 2021 Jul 15;16(7):e0254176. doi: 10.1371/journal.pone.0254176 (PMC8282069; doi:10.1371/journal.pone.0254176)
Supplement: S13 Table — (DOCX) [file pone.0254176.s016.docx]

| **S13 Table.** **Correlations between responses to Section 4: Listening to Music including indicators of s*elf-reported musical ability* and *music listening*** | | | | | | | |
| --- | --- | --- | --- | --- | --- | --- | --- |
|  | 1 | 2 | 3 | 4 | 5 | 6 | 7 |
| 1. Recorded music hours per week | - |  |  |  |  |  |  |
| 2. Concerts/gigs per year | .311** | - |  |  |  |  |  |
| 3. Parents sang in the home | .141** | .165** | - |  |  |  |  |
| 4. Clap hands to music | .222** | .133** | .265** | - |  |  |  |
| 5. Dance in time to music | .148** | .139** | .192** | .571** | - |  |  |
| 6. Sing a melody in tune | .148** | .118* | .306** | .498** | .481** | - |  |
| 7. Listening to music important | .442** | .413** | .270** | .392** | .303** | .367** | - |
| 8. Strong emotional response to music | .350** | .353** | .263** | .429** | .414** | .396** | .710** |
| Correlations are non-parametric Spearman’s rho. Correlations highlighted in light and darker grey show correlations between indicators of *music listening* and *self-reported musical ability*, respectively. | | | | | | | |
