# Supplementary material for: The Edinburgh Lifetime Musical Experience Questionnaire (ELMEQ): Responses and non-musical correlates in the Lothian Birth Cohort 1936
Source: PLoS One. 2021 Jul 15;16(7):e0254176. doi: 10.1371/journal.pone.0254176 (PMC8282069; doi:10.1371/journal.pone.0254176)
Supplement: S14 Table — (DOCX) [file pone.0254176.s017.docx]

| **S14 Table. Childhood correlates of musical experience.** | | | | |
| --- | --- | --- | --- | --- |
| Experience | Covariate | *β* | 95% CI | *p* |
| *Playing an instrument* | Sex | 0.104 | -0.148, 0.355 | 0.420 |
|  | Age 11 cog ability | 0.074 | -0.053, 0.202 | 0.255 |
|  | Childhood environment | **-0.252** | -0.407, -0.097 | 0.001 |
|  | Years of education | **0.165** | 0.021, 0.310 | 0.025 |
|  | Father’s social class | -0.085 | -0.218, 0.049 | 0.213 |
| *Singing* | Sex | **0.587** | 0.342,0.832 | <0.001 |
|  | Age 11 cog ability | **0.183** | 0.042, 0.324 | 0.011 |
|  | Childhood environment | -0.066 | -0.218, 0.087 | 0.399 |
|  | Years of education | **0.206** | 0.066, 0.346 | 0.004 |
|  | Father’s social class | -0.027 | -0.176, 0.123 | 0.728 |
| *Music listening* | Sex | **0.541** | 0.245,0.838 | <0.001 |
|  | Age 11 cog ability | **0.213** | 0.046, 0.380 | 0.012 |
|  | Childhood environment | -0.006 | -0.183, 0.170 | 0.942 |
|  | Years of education | 0.009 | -0.188, 0.206 | 0.927 |
|  | Father’s social class | 0.011 | -0.154, 0.175 | 0.897 |
| *Self-reported musical ability* | Sex | **0.372** | 0.13,0.613 | 0.003 |
|  | Age 11 cog ability | 0.091 | -0.049, 0.23 | 0.205 |
|  | Childhood environment | 0.023 | -0.133, 0.18 | 0.769 |
|  | Years of education | -0.033 | -0.183, 0.118 | 0.671 |
|  | Father’s social class | -0.023 | -0.161, 0.114 | 0.742 |

Estimates in bold are statistically significant (*p* < 0.05). Covariates are treated as continuous variables. Sex coded as 0= male, 1 = female. Lower scores for childhood environment indicate a lower level of deprivation. Lower scores for Father’s social class indicate a more professional occupation. The table shows standardized parameter estimates. For binary covariates a different type of standardization is used which can be interpreted as a change in the dependent variable in standard deviation units when the binary covariate changes from zero to one.
