# Supplementary material for: The Edinburgh Lifetime Musical Experience Questionnaire (ELMEQ): Responses and non-musical correlates in the Lothian Birth Cohort 1936
Source: PLoS One. 2021 Jul 15;16(7):e0254176. doi: 10.1371/journal.pone.0254176 (PMC8282069; doi:10.1371/journal.pone.0254176)
Supplement: S15 Table — (DOCX) [file pone.0254176.s018.docx]

| **S15 Table. Older age correlates of musical experience.** | | | | |
| --- | --- | --- | --- | --- |
| Experience | Covariate | *β* | 95% CI | *p* |
| *Playing an instrument* | **Social class** | -0.291 | -0.427, -0.155 | <0.001 |
|  | Environmental quality | 0.006 | -0.146, 0.158 | 0.938 |
|  | Activities of daily living | -0.001 | -0.15, 0.149 | 0.994 |
|  | History of diabetes | -0.081 | -0.493, 0.331 | 0.700 |
|  | History of cardiovascular disease | -0.135 | -0.405, 0.135 | 0.326 |
|  | History of stroke | 0.075 | -0.319, 0.469 | 0.709 |
|  | History of cancer | 0.162 | -0.144, 0.467 | 0.299 |
|  | History of arthritis | -0.097 | -0.366, 0.172 | 0.480 |
|  | History of Parkinson’s | 0.081 | -1.624, 1.787 | 0.926 |
|  | History of Dementia | -0.512 | -1.639, 0.614 | 0.373 |
|  | Sex | 0.041 | -0.228, 0.310 | 0.764 |
| *Singing* | **Social class** | -0.228 | -0.373, -0.084 | 0.002 |
|  | Environmental quality | 0.024 | -0.129, 0.178 | 0.757 |
|  | Activities of daily living | -0.006 | -0.148, 0.137 | 0.937 |
|  | History of diabetes | -0.369 | -0.84,0.101 | 0.124 |
|  | History of cardiovascular disease | 0.045 | -0.238,0.327 | 0.757 |
|  | History of stroke | 0.260 | -0.101,0.621 | 0.158 |
|  | History of cancer | 0.138 | -0.193,0.468 | 0.415 |
|  | **History of arthritis** | 0.267 | 0.004,0.531 | 0.047 |
|  | History of Parkinson’s | -0.124 | -2.093,1.845 | 0.902 |
|  | History of Dementia | 0.193 | -0.752,1.138 | 0.689 |
|  | **Sex** | 0.471 | 0.208,0.733 | <0.001 |
| *Music listening* | Social class | -0.114 | -0.282, 0.054 | 0.184 |
|  | Environmental quality | 0.050 | -0.102, 0.202 | 0.522 |
|  | Activities of daily living | 0.039 | -0.138, 0.215 | 0.668 |
|  | History of diabetes | -0.241 | -0.669,0.187 | 0.270 |
|  | History of cardiovascular disease | -0.099 | -0.421,0.223 | 0.546 |
|  | History of stroke | -0.101 | -0.559,0.357 | 0.665 |
|  | History of cancer | 0.175 | -0.212,0.561 | 0.376 |
|  | History of arthritis | -0.037 | -0.364,0.289 | 0.823 |
|  | History of Parkinson’s | -0.271 | -3.172,2.63 | 0.855 |
|  | History of Dementia | -0.489 | -1.229,0.25 | 0.195 |
|  | **Sex** | 0.477 | 0.163,0.792 | 0.003 |
| *Self-reported musical ability* | Social class | -0.074 | -0.208, 0.06 | 0.279 |
|  | Environmental quality | -0.058 | -0.184, 0.069 | 0.371 |
|  | **Activities of daily living** | -0.199 | -0.329, -0.069 | 0.003 |
|  | History of diabetes | 0.099 | -0.267,0.465 | 0.596 |
|  | History of cardiovascular disease | 0.124 | -0.137,0.385 | 0.352 |
|  | History of stroke | -0.276 | -0.642,0.091 | 0.140 |
|  | History of cancer | -0.157 | -0.475,0.162 | 0.336 |
|  | History of arthritis | 0.159 | -0.103,0.421 | 0.233 |
|  | History of Parkinson’s | -0.321 | -1.594,0.952 | 0.621 |
|  | History of Dementia | 0.008 | -0.661,0.678 | 0.980 |
|  | **Sex** | 0.415 | 0.159,0.672 | 0.001 |

Estimates in bold are statistically significant (*p* < 0.05). Covariates are treated as continuous variables. Lower scores for social class indicate a more professional occupation. Higher scores on environmental quality indicate better quality. Lower scores on activities of daily living indicate fewer constraints. History of disease is coded as 1 = yes, 0 = no. The table shows standardized parameter estimates. For binary covariates a different type of standardization is used which can be interpreted as a change in the dependent variable in standard deviation units when the binary covariate changes from zero to one.
