# Supplementary material for: The Edinburgh Lifetime Musical Experience Questionnaire (ELMEQ): Responses and non-musical correlates in the Lothian Birth Cohort 1936
Source: PLoS One. 2021 Jul 15;16(7):e0254176. doi: 10.1371/journal.pone.0254176 (PMC8282069; doi:10.1371/journal.pone.0254176)
Supplement: S16 Table — (DOCX) [file pone.0254176.s019.docx]

| **S16 Table. Personality correlates of musical experience.** | | | | |
| --- | --- | --- | --- | --- |
| Experience | Covariate | *β* | 95% CI | *p* |
| *Playing an instrument* | Extraversion | 0.082 | -0.076, 0.24 | 0.307 |
|  | Agreeableness | 0.000 | -0.159, 0.159 | 1.000 |
|  | Conscientiousness | 0.034 | -0.124, 0.192 | 0.673 |
|  | Emotional stability | -0.052 | -0.205, 0.101 | 0.503 |
|  | **Openness to experience** | 0.160 | 0.029, 0.292 | 0.017 |
|  | Sex | 0.044 | -0.236,0.324 | 0.759 |
| *Singing* | Extraversion | 0.069 | -0.086, 0.224 | 0.382 |
|  | Agreeableness | -0.098 | -0.275, 0.08 | 0.280 |
|  | Conscientiousness | 0.068 | -0.079, 0.215 | 0.365 |
|  | Emotional stability | -0.116 | -0.26, 0.028 | 0.115 |
|  | **Openness to experience** | 0.187 | 0.048, 0.325 | 0.008 |
|  | **Sex** | 0.548 | 0.27,0.825 | <0.001 |
| *Music listening* | **Extraversion** | 0.205 | 0.023, 0.388 | 0.028 |
|  | Agreeableness | -0.001 | -0.184, 0.182 | 0.989 |
|  | Conscientiousness | -0.068 | -0.229, 0.093 | 0.407 |
|  | Emotional stability | -0.009 | -0.177, 0.159 | 0.919 |
|  | **Openness to experience** | 0.207 | 0.041, 0.373 | 0.014 |
|  | **Sex** | 0.477 | 0.148,0.806 | 0.004 |
| *Self-reported musical ability* | **Extraversion** | 0.240 | 0.095, 0.385 | 0.001 |
|  | Agreeableness | 0.026 | -0.117, 0.168 | 0.725 |
|  | Conscientiousness | 0.095 | -0.043, 0.233 | 0.179 |
|  | Emotional stability | -0.040 | -0.17, 0.089 | 0.540 |
|  | Openness to experience | 0.067 | -0.065, 0.199 | 0.319 |
|  | **Sex** | 0.268 | 0.017,0.52 | 0.037 |

Estimates in bold are statistically significant (*p* < 0.05). The table shows standardized parameter estimates.
