# Supplementary material for: The Edinburgh Lifetime Musical Experience Questionnaire (ELMEQ): Responses and non-musical correlates in the Lothian Birth Cohort 1936
Source: PLoS One. 2021 Jul 15;16(7):e0254176. doi: 10.1371/journal.pone.0254176 (PMC8282069; doi:10.1371/journal.pone.0254176)
Supplement: S1 File — (DOCX) [file pone.0254176.s020.docx]

Edinburgh Lifetime Musical Experience Questionnaire (ELMEQ)

Section 1: Musical instruments

|  | **Questions** | **Answers (please circle your answer where appropriate)** | | | | | | | | | | | | | | | | | |
| --- | --- | --- | --- | --- | --- | --- | --- | --- | --- | --- | --- | --- | --- | --- | --- | --- | --- | --- | --- |
| 1. | Have you ever learned to play a musical instrument? | Yes / No (*If ‘No’ please go straight to Section 2: Singing)* | | | | | | | | | | | | | | | | | |
|  | If **yes**, on **how many** musical instruments could you comfortably perform a short piece of music (either in the past or currently)? | 0 | | 1 | | | | 2 | | | 3 | | | | 4 | | | 5 or more | |
| 2. | Please list the names of these instruments **starting with the highest level of expertise** | Instrument | | | | | Age began | | | | Years played | | | Level reached (exam grade or beginner/intermediate /advanced/semi-professional /professional) | | | | | |
| 3. | How old were you when you started to learn your **first** musical instrument? | 0-3 | | | 4-5 | | | | 6-7 | | | | 8-11 | | | | 12 or older | | |
| 4. | Did you have any formal instrumental training (e.g. school lessons, private lessons or conservatoire training)? | Yes / No | | | | | | | | | | | | | | | | | |
|  | If **yes**, **how many years** of formal musical training did you complete? | 1 or less | | | 2-5 | | | | 6-10 | | | | 11-20 | | | | 21+ | | |
| 5. | Do you still play a musical instrument currently? | Yes / No | | | | | | | | | | | | | | | | | |
|  | If **no**, at approximately **what age** did you stop playing? | Age stopped playing: _____ | | | | | | | | | | | | | | | | | |
|  | If **yes**, approximately **how many hours** do you currently play per week, on average? | 1 or less | | | 2-3 | | | | 4-6 | | | | 7-13 | | | | 14+ | | |
| 6. | For **how many years** of your life in total would you say that you regularly (e.g. at least once a week) played a musical instrument of some kind (not including breaks)? | 5 or less | | | 6-10 | | | | 11-20 | | | | 21-40 | | | | 41+ | | |
| 7. | During the years you played your instrument(s) regularly, **how many hours** per week did you practise, on average (not including group practise)? | 1 or less | | | 2-3 | | | | 4-6 | | | | 7-13 | | | | 14+ | | |
| 8. | Overall, what would you say is the **highest level** of musical performance that you reached? | Beginner | | | Intermediate | | | | Advanced | | | | Semi-professional | | | | Professional | | |
| 9. | Have you ever played with a band, ensemble or orchestra? | Yes / No (*If ‘No’ please go to question 10)* | | | | | | | | | | | | | | | | | |
|  | If **yes**, for **how many years** of your life did you play with a musical group (not including breaks)? | 5 or less | | | 6-10 | | | | 11-20 | | | | 21-40 | | | | 41+ | | |
|  | If **yes**, during the time that you played with a group/groups, for how many **hours per week** did you practise, rehearse, play or perform **together**, on average? | 1 or less | | | 2-3 | | | | 4-6 | | | | 7-13 | | | | 14+ | | |
| 10 | During the time that you played your musical instrument **either solo or with a group**, did you ever practise, rehearse, play or perform for 7 or more hours per week? | Yes / No (*If ‘No’ please go to question 11)* | | | | | | | | | | | | | | | | | |
|  | If yes, during which decades of your life did you regularly practise, rehearse, play or perform **for 7 or more hours per week?** (circle all that apply) | age  0-9 | age  10-19 | | | age  20-29 | | age  30-39 | | age  40-49 | | age  50-59 | | | age  60-69 | age  70-79 | | | age  80-89 |
| 11. | When practising, rehearsing, playing and performing, did/do you regularly: | Read from a musical score? Yes / No  Learn and play pieces by ear? Yes / No  Improvise? Yes / No | | | | | | | | | | | | | | | | | |

Section 2: Singing

|  | **Questions** | **Answers (please circle your answer where appropriate)** | | | | |
| --- | --- | --- | --- | --- | --- | --- |
| 1. | Have you ever sung in a group (choir, band, or another kind of ensemble?) | Yes / No (*If ‘No’ go straight to Question 6: Solo vocal training)* | | | | |
| 2. | **How old** were you when you started to sing in a group (choir, band or ensemble)? | 0-3 | 4-5 | 6-7 | 8-11 | 12 or older |
| 3. | For **how many years** in total have you sung in a group (choir/band or ensemble) not including breaks? | Less than 5 | 5-10 | 11-20 | 21-40 | 41+ |
| 4. | During the years you sang in a group (choir/band or ensemble), for **how many hours per week** did you practise, rehearse or perform together, on average? | 1 or less | 2-3 | 4-6 | 7-13 | 14+ |
| 5. | Do you still sing in a group currently? | Yes / No | | | | |
|  | If **no**, at approximately **what age** did you stop singing in a group? | Age stopped singing in a group: _____ | | | | |
|  | If **yes**, approximately **how many hours** do you currently sing per week, on average? | 1 or less | 2-3 | 4-6 | 7-13 | 14+ |
| 6. | Did you have any formal solo vocal training (e.g. school lessons, private lessons or conservatoire training)? | Yes / No | | | | |
| 7. | If yes, **how many years** of formal solo vocal training did you complete? | 1 or less | 2-5 | 6-10 | 11-20 | 21+ |

Section 3: Reading Musical Notation

|  | **Questions** | **Answers (please circle your answer where appropriate)** | | | | |
| --- | --- | --- | --- | --- | --- | --- |
| 1. | Have you ever learned to read any kind of music notation? | Yes / No (*If ‘No’ please go straight to Section 4: Listening to Music)* | | | | |
| 2. | Can you read:  (please **circle all that apply**) | Treble Clef  Bass clef  Alto clef  Guitar tab  Percussion  Chord symbols (e.g. C, Am, G7) | | | | |
| 3. | What is the **highest level** of sight-reading expertise that you have reached? | Beginner | Intermediate | Advanced | Semi-professional | Professional |

Section 4: Listening to Music

|  | **Questions** | **Answers (please circle your answer where appropriate)** | | | | |
| --- | --- | --- | --- | --- | --- | --- |
| 1. | **On average, over the course of your life**, how many **hours per week** would you say that you have actively listened to recorded music such as via the stereo, radio, or other device, by choice? | 1 or less | 2-3 | 4-6 | 7-13 | 14+ |
| 2. | **On average, over the course of your life**, how many live music concerts or gigs do you/have you attended per year? | 1 or less | 2-6 | 7-24 | 25-49 | 50+ |
| 3. | How easy do you find it to clap your hands in time to music? | Very difficult | Difficult | Not sure | Easy | Very easy |
| 4. | How easy do you find it to dance in time to music? | Very difficult | Difficult | Not sure | Easy | Very easy |
| 5. | How easy do you find it to sing a melody in tune? | Very difficult | Difficult | Not sure | Easy | Very easy |
| 6. | As a child, how often do you think your parents sang lullabies, nursery rhymes, or other songs in the home? | Never | Rarely | Sometimes | Regularly | Every day |
| 7. | How important has listening to music been to you over the course of your life? | Not at all  important | Not very important | Not sure | Quite important | Very important |
| 8. | Would you say that you have strong emotional responses to music? | Not at all | Not really | Not sure | Quite strong | Very strong |
| 9. | Do you have any other musical experience you would like to tell us about, or any further comments? |  | | | | |

Thank you for completing this questionnaire!

Highlighted questionnaire items were used in the current study as indicators of the latent variables *Playing a musical instrument* (green), *Singing* (blue), *Listening to Music* (pink) and *Self-reported musical ability* (purple).

**Updates to the ELMEQ that was administered to LBC1936 participants**

Section 1: Musical Instruments

1. Removed option for fourth instrument from question 2 (Please list the names of these instruments). Removed to save space, no participants reported playing more than 3 musical instruments.
2. Example responses (beginner/intermediate/advanced/semi-professional/professional) for level reached added to question 2.
3. “not including group practice” added to question 7 to exclude any group practice.
4. The phrase “not including breaks” added to the second item of question 9, to be consistent with equivalent questions about years playing solo and singing.
5. “rehearse or perform” changed to “practice, rehearse, play or perform” to include informal ways of playing music not classed as rehearsal or performance. These changes were made to questions 9, 10, and 11.
6. Additional item added to question 10: “During the time that you played your musical instrument either solo or with a group, did you ever practice, rehearse or perform for 7 or more hours per week?” This item was added to identify participants for whom the next question “during which decades of your life did you regularly practise, rehearse or perform for 7 or more hours per week?” was not applicable.

Section 2: Singing

1. For question 1, changed “If ‘No’ go straight to Section 3” to “If ‘No’ go straight to Question 6: Solo vocal training” to capture information about participants with no experience singing in a group but with experience of solo vocal training.
2. The phrase “on average” was added to question 4, to be consistent with the equivalent question for musical instruments.
3. An additional question (question 5 on the updated version) was added to capture information regarding current singing.

Section 4: Listening to Music

1. Question 3 changed from “How easy do you find it to clap your hands to music” to “How easy do you find it to clap your hands *in time* to music”, for clarity.
